# Anlotinib Combined with Toripalimab as Second-Line Therapy for Advanced, Relapsed Gastric or Gastroesophageal Junction Carcinoma

**DOI:** 10.1093/oncolo/oyac136

**Published:** 2022-07-20

**Authors:** Man Jiang, Chuantao Zhang, Yabin Hu, Tianjun Li, Guangjie Yang, Guanqun Wang, Jingjuan Zhu, Changfeng Shao, Helei Hou, Na Zhou, Kewei Liu, Xiaochun Zhang

**Affiliations:** Cancer Precision Medical Center, The Affiliated Hospital of Qingdao University, Qingdao University, Qingdao, People’s Republic of China; Cancer Precision Medicine Laboratory, The Affiliated Hospital of Qingdao University, Qingdao University, Qingdao, People’s Republic of China; Cancer Precision Medical Center, The Affiliated Hospital of Qingdao University, Qingdao University, Qingdao, People’s Republic of China; Department of Radiology, The Affiliated Hospital of Qingdao University, Qingdao University, Qingdao, People’s Republic of China; Cancer Precision Medical Center, The Affiliated Hospital of Qingdao University, Qingdao University, Qingdao, People’s Republic of China; Department of Nuclear Medicine, The Affiliated Hospital of Qingdao University, Qingdao University, Qingdao, People’s Republic of China; Pathology Department, The Affiliated Hospital of Qingdao University, Qingdao University, Qingdao, People’s Republic of China; Cancer Precision Medical Center, The Affiliated Hospital of Qingdao University, Qingdao University, Qingdao, People’s Republic of China; Department of Transfusion, The Affiliated Hospital of Qingdao University, Qingdao, People’s Republic of China; Cancer Precision Medical Center, The Affiliated Hospital of Qingdao University, Qingdao University, Qingdao, People’s Republic of China; Cancer Precision Medicine Laboratory, The Affiliated Hospital of Qingdao University, Qingdao University, Qingdao, People’s Republic of China; Cancer Precision Medical Center, The Affiliated Hospital of Qingdao University, Qingdao University, Qingdao, People’s Republic of China; Cancer Precision Medicine Laboratory, The Affiliated Hospital of Qingdao University, Qingdao University, Qingdao, People’s Republic of China; Cancer Precision Medical Center, The Affiliated Hospital of Qingdao University, Qingdao University, Qingdao, People’s Republic of China; Cancer Precision Medical Center, The Affiliated Hospital of Qingdao University, Qingdao University, Qingdao, People’s Republic of China; Cancer Precision Medicine Laboratory, The Affiliated Hospital of Qingdao University, Qingdao University, Qingdao, People’s Republic of China

**Keywords:** anlotinib, toripalimab, second-line therapy, gastric or gastroesophageal junction carcinoma, tumor microenviroment

## Abstract

Our study aimed to explore the efficacy and safety of anlotinib–toripalimab combination therapy as a second-line treatment for advanced relapsed gastric or gastroesophageal junction carcinoma (GC/GEJC). In this single arm, single-center extension clinical trial, patients with advanced relapsed GC/GEJC received toripalimab (240 mg, intravenously over 60 minutes, once every 2 weeks) plus anlotinib (12 mg/day, orally, 2 weeks on and 1 week off, every 3 weeks) as second-line therapy. There were 29 patients who achieved partial response, and the ORR was 32.3% (95% CI, 26.6%-38.5%). Grade 3 treatment-related adverse events (TRAEs) were recorded in 7 participants (11.3%), all of which were manageable. The PFS and OS were 4.0 and 11.1 months, respectively. Patients with programmed death-ligand 1 (PD-L1) positive expression showed numerically longer OS than the negative ones although the difference was not significantly. The tumor mutational burden-high (TMB-H) group showed a significantly better OS (*P* = .05) than the TMB-Low (TMB-L) group. Next-generation sequencing (NGS) revealed that fibroblast growth factor receptor 2 (*FGFR2)* mutations positively correlated with target lesion reduction (odds ratio [OR] = 0.14; *P = .*02). The new regimen increased tumor-infiltration of CD8^+^ T and CD3^+^ T cells. Furthermore, a patient-derived organoid (PDO) study indicated that anlotinib could promote an immune-supportive tumor microenvironment. As conclusion, the anlotinib-toripalimab combination showed promising efficacy and favorable safety as a second-line treatment for advanced, relapsed GC/GEJC. The PD-L1 expression, TMB, and *FGFR2* mutation are potential biomarkers for predicting the efficacy of this regimen (ClinicalTrials.gov registration number: NCT04713059).

## Teaser

This article explores the safety and efficacy of anlotinib combined with toripalimab as second-line therapy for Chinese patients with advanced, relapsed gastric or gastroesophageal junction carcinoma.

**Implications for Practice**

The anlotinib–toripalimab combination showed promising efficacy and favorable safety as a second-line treatment for advanced, relapsed gastric, or gastroesophageal junction carcinoma. PD-L1 expression, tumor mutational burden, and *FGFR2* mutation status can be biomarkers for predicting the efficacy of this novel regimen.

## Introduction

Gastric or gastroesophageal junction carcinoma (GC/GEJC) is the third leading cause of cancer-related death worldwide and the second deadliest malignancy in China.^1^ More than 80% of patients are diagnosed at an advanced stage of disease, with a 5-year survival rate of <20%.^2^ The prolonged benefit of first-line chemotherapy for advanced GC/GEJC is limited, and approximately 50% of patients are eligible to receive second-line treatment.^2^ Currently, the ramucirumab plus paclitaxel regimen is recommended as second-line therapy for advanced or metastatic gastric cancer. However, its efficacy is unsatisfactory, with an objective response rate (ORR) of 17%–28% and progression-free survival (PFS) of 2–4 months.^3^ Due to the poor prognosis, there is an unmet need for new effective second-line treatment options for advanced relapsed GC/GEJC.

Anti-programmed death-1 (PD-1) antibodies have demonstrated a durable response and prolonged survival in patients with multiple types of tumors. Recently, 2 anti-PD-1 monoclonal antibodies, nivolumab and pembrolizumab, have been successively approved by the US Food and Drug Administration (FDA) to incorporate into first-line chemotherapy for GC/GEJC.^4,5^ Although selected patients with a high programmed death-ligand 1 (PD-L1) combined positive score (CPS) have demonstrated a survival benefit from anti-PD-1 or anti-PD-L1 treatment, in non-selected patients the response rates of anti-PD-1 or PD-L1 therapies are less than 20%.^3^ The search for an optimal combination regime that enhances PD-1 inhibitor-based therapy is currently a major focus of research.

Previous studies have indicated that antiangiogenic agents, such as tyrosine kinase inhibitors (TKIs), demonstrate synergistic effects with anti-PD-1 antibodies by modulating the tumor microenvironment (TME).^6,7^ Abnormal tumor vessels reduce the infiltration of T cells into the TME, promoting the accumulation of suppressive immune cells. Thus, normalization of tumor vessels increases the recruitment and infiltration of T cells, thereby improving the immune effect and enhancing the anti-tumor effect. While hundreds of phase I/II clinical trials of antiangiogenics plus immunotherapy have been reported, but the best “combination synergy” for GC/GEJC, has not yet been found.^8-11^ Fukuoka et al reported regorafenib plus nivolumab had a manageable safety profile and showed encouraging antitumor activity in patients with gastric and colorectal cancer, which was the very first documentation of the combinations in gastric cancer.^8^ In another previous clinical trial, lenvatinib combined with pembrolizumab administered to 29 Japanese gastric patients showed an ORR of 69% as first- or second-line treatment for GC/GEJC and a PFS of 7.1 months.^10^ However, the high rate of serious treatment-related adverse events (TRAEs) (grade 3 or 4 TRAEs occurred in 48% of patients) limited the clinical application of the regimen. In another clinical trial involving a Chinese population, apatinib combined with camrelizumab as a second-line therapy for GC/GEJC had a slightly lower incidence of grade 3 or 4 TRAEs (60.6%), but the ORR was only 16% and PFS was only 1.9 months.^11^ Based on these results, TKIs show early signs of promising efficacy on improving the response rate of PD-1 blockade for GC/GEJC. However, novel combinations with high antitumor activity and acceptable safety are still being explored.^12,13^

Anlotinib, an oral multi-targeting TKI that targets to vascular endothelial growth factor receptor (VEGFR), fibroblast growth factor receptor (FGFR), platelet-derived growth factor receptors (PDGFR), and c-Kit,^14-16^ has good safety and efficacy in many advanced refractory solid tumors, including GC/GEJC.^14,17^ Compared with apatinib, which was the first small-molecular inhibitor approved for advanced gastric cancer treatment in China, anlotinib has shown more delayed drug resistance development and fewer side effects.^17^ It has been reported that anlotinib can reduce immunosuppressive cytokines and expression of inhibitory checkpoints in cluster of differentation (CD)8^+^ T cells, as well as potentially improve immunotherapy efficacy by normalizing the tumor microenvironment.^18^ Furthermore, a prospective study of anlotinib combined with anti-PD-1 antibody therapy in patients with advanced refractory solid tumors indicated that the combination of anlotinib and anti-PD-1 antibodies significantly increased the proportion of CD4^+^ T cells and CD8^+^ T cells in the TME, decreased the proportion of myeloid-derived suppressor cells (MDSCs), and improved the effect of immunotherapy.^18^ Toripalimab is a novel anti-PD-1 antibody that binds to the FG loop of the PD-1 receptor, in contrast to well-described nivolumab and pembrolizumab, which bind to the N-terminal loop and the C’D loop of PD-1, respectively.^19^ In an in vitro antigen recall study, toripalimab showed an efficacy in promoting T-cell proliferation that was similar to that of nivolumab. Furthermore, compared with nivolumab, toripalimab could induce interferon-ϒ cytokine production much more strongly than nivolumab, indicating that toripalimab could potentially have a more positive response rate than nivolumab.^19^ In previous clinical trials, toripalimab showed a well-manageable safety profile.^20-22^ In a Chinese population-based phase I study of toripalimab for refractory malignant solid tumors, the 25 enrolled patients showed no TRAEs of grade 3 or higher.^20^ Another phase Ib clinical trial showed that toripalimab monotherapy had an almost equivalent ORR to pembrolizumab or nivolumab (11.6%, 11.2%, and 12.1% for pembrolizumab, nivolumab, and toripalimab, respectively) and tolerable toxicity in the treatment of recurrent or metastatic GC/GEJC.^22-24^ On the basis of the findings above, herein we explored the safety and efficacy of anlotinib combined with toripalimab for the first time as second-line therapy for Chinese patients with advanced, relapsed GC/GEJC and investigated the predictive biomarker for this novel regimen.

## Methods

### Study Design and Participants

A single-armed, single-center, investigator-initiated, open-label, and exploratory trial was conducted at the Cancer Precision Medical Center, Affiliated Hospital of Qingdao University (Qingdao, China). The Institutional Review Board of the Affiliated Hospital of Qingdao University approved the study protocol (no. QYFYKYLL471311920). Prior to enrollment, all participants provided their written informed consent in accordance with the Declaration of Helsinki (as revised in 2013). The subgroup analyses by PD-L1 expression, microsatellite instability (MSI), and tumor mutational burden (TMB) were prespecified.

Eligible patients were 18 years or older with pathologically diagnosed advanced, relapsed GC/GEJC adenocarcinoma and had only 1 previous systemic therapy regimen. All enrolled patients had experienced either disease progression during first-line therapy, recurrence in the first year after definitive therapy, or intolerance to first-line therapy. Patients had ≥1 measurable disease at baseline as per the Response Evaluation Criteria in Solid Tumors, version 1.1 (RECIST v. 1.1). Eastern Cooperative Oncology Group (ECOG) performance status score of 0 to 1, and adequate organ and bone marrow function. Exclusion criteria included history of autoimmune disease, ongoing infections, or prior CTLA-4 or PD-L1 checkpoint blockade immunotherapy (Supplementary Material).

### Treatments and Follow-up

Participants received anlotinib (12 mg/day, orally; 2 weeks of treatment followed by 1 week off) combined with toripalimab (240 mg, via intravenous-drip over 60 minutes, once every 3 weeks; Supplementary Table S1). Treatment continued until the investigators determined that the patients had developed tolerance to the treatment or when disease progression was confirmed. During administration, the investigator adjusted the doses based on the study protocol (Supplementary Tables S2-S7). Treatment was terminated if it was necessary to reduce the dose by more than 2 increments. Supplementary Table S3 shows the recommended delay in medication administration and dosage changes when therapy-related toxicity occurred (values for platelet count decrease, hemorrhage, liver dysfunction, and proteinuria can be found in Supplementary Tables S4-S8). Investigators assessed therapeutic efficacy based on CT imaging according to RECIST v.1.1 every 2 treatment cycles (3 weeks/cycle) in the first 6 cycles and every 4 treatment cycles thereafter. Laboratory evaluations, including hematology, blood chemistry, and magnesium level, were carried out on day 1 (before drug administration) of each treatment cycle. Participants were evaluated for toxicity and adverse events on the same schedule. Echocardiography was performed on day 1 of cycle 3 (before drug administration) and every 4 cycles after that.

### Endpoints

The primary endpoint was ORR, defined as the proportion of patients who achieved an overall response (complete response [CR] or partial response [PR] according to RECIST version 1.1) and safety. Secondary endpoints included PFS, determined as the time from the start of toripalimab and anlotinib treatment to disease progression or death. The correlation of potential biomarkers with clinical efficacy was examined as an exploratory endpoint.

### EBV Status

In situ hybridization (ISH) staining for EBV-encoded RNA (EBER) was used to detect EBV states. We used antibodies against EBNA1 (Santa Cruz, Heidelberg, 1EB12) and LMP-1 (Abcam, D24G). ISH for EBER was carried out in each sample on 5 μm-thick sections as previously described.^25^

### Expression of PD-L1

Tumor biopsies were obtained before treatment. PD-L1 staining was scored as previously reported.^26,27^ Immunohistochemical (IHC) labeling was performed using antibodies against PD-L1 (clone SP263; Roche Tissue Diagnostics, Oro Valley, AZ, USA) and PD-1 (clone NAT; Abcam, Cambridge, UK). Positive expression of PD-L1 in this study was defined as a CPS of 1 or more as previously described.^26,28^

### Next-generation Sequencing

Next-generation sequencing (NGS) was performed using an Onco Screen Plus kit (Burning Rock Biotech, Guangzhou, China), with a panel consisting of 520 cancer-related genes spanning 1.64 Mb of the human genome on the NextSeq platform (Illumina, San Diego, CA, USA). The TMB was determined by analyzing somatic mutations per megabase (Mb). A TMB of ≥ 10 mutations/Mb was defined as TMB-High (TMB-H). Patients with a TMB of < 10 mutations/Mb were defined as TMB-Low (TMB-L). MSI status was determined by NGS assay.^27^

### TME Analysis

The TME of the selected patient (participant G20) with negative PD-L1 expression and none of the examined gene mutations in the 520-NGS panel mentioned above was analyzed. Multiplex staining and multispectral imaging with the PANO 7-plex IHC kit (cat 0004100100; Panovue, Beijing, China) were used to identify cell subsets expressing CD3, CD8, PD-1, and PD-L1 of archival tissues, which were used as TME markers.

### Organoid Study

Fresh tumor tissue samples of the selected patient mentioned above were obtained by endoscopic biopsy. The tissue was washed with ice-cold phosphate-buffered saline (PBS) at least 10 times, and subsequently digested with Liberase (TH grade; Roche Life Science) for 60 minutes at 37ºC, with vigorous pipetting every 15 minutes. The remaining fragments were additionally treated with Tryp LE Express (Invitrogen) at 37ºC for 20 minutes. The supernatant was collected and centrifuged at 200 × *g* for 3 minutes at 4ºC. The cell pellet was suspended with Matrigel (growth factor reduced; BD Biosciences) and cultured in drops of basement membrane extract (BME; Amsbio, Cambridge, MA, USA),^29^ with the medium refreshed every 2 days. We performed IHC to verify the characteristics of the organoids. At 5 days after organoid trypsinization, the patient-derived organoids (PDOs) were divided into 4 groups: control, anlotinib-50 (50 µg/mL), anlotinib-100 (100 µg/mL), and anlotinib-200 (200 µg/mL). The average diameter of the PDOs was measured as the basis for efficacy evaluation. Tumor cell viability was assessed on a fluorescence spectrophotometer (Hitachi F2000 fluorescence spectrophotometer, Hitachi, Tokyo, Japan). Therapeutic efficacy was compared among the different doses. The Search Tool for the Retrieval of Interacting Genes/Proteins (STRING database http://string-db.org)^30^ was used to study the interaction of anlotinib targets and immunotherapy. The expression levels of *STAT1*, *STAT3*, *phosphorylated (p)STAT1*, and *pSTAT3* were preliminarily measured by western blotting.

### Data Analysis

The sample size was estimated based on an assumed ORR of 10% failure for the first-line systemic treatments and an ORR of 30% for combination therapy of anlotinib plus toripalimab. This study was designed to have at least 95% (1-β) power at a 2-sided significance level of 0.5% to reject the null hypothesis of a proportion of patients with an ORR of 10% or less in this population. Considering an assumed drop-out rate of 20%, we planned to recruit 62 patients (PASS 15.0.5). It was calculated that if objective response (ie, CR or PR) was achieved in 18 of the 62 participants, the treatment could be recommended for further efficacy evaluation.^31^ Survival analysis (ie, PFS and OS) was performed using the Kaplan–Meier method, and the log-rank test was used to evaluate the differences between groups. The Cox proportional hazards model was used to calculate hazard ratios (HRs) with their corresponding 95% CIs. The safety population encompassed all participants, including 3 who later dropped out before the first cycle of treatment had completed. Data were summarized as numbers (proportions) for categorical variables and mean ± standard error for continuous variables. Categorical groups were explored for biomarker evaluation, with the variable distribution considered to evaluate the association with response and/or survival. A *P-*value threshold of 0.05 was used to indicate statistical significance for all evaluations. Analyses were conducted using SPSS 20 software (IBM Corp., Armonk, NY, USA) or Graph Pad Prism 9 (Graph Pad Software, Inc., San Diego, CA, USA). In the organoid study, 3 independent experiments were performed. The *P*-values were used to assess statistical significance.

## Results

### Patient Population and Baseline Characteristics

At the data cutoff (31 December, 2020), 62 patients were enrolled in this study. Trial details are listed in Supplementary Table S1, and the baseline characteristics of the participants are presented in Table 1. Four patients had disease progression during first-line therapy, and 2 patients were intolerant to first-line therapy. A total of 56 of 62 participants experienced recurrence in the first year after definitive therapy. The median age of participants was 62 years, and 37 (59.7%) were male. The mean body mass index (BMI) was 19.4 ± 3.4 kg/m^2^. A total of 12 participants (19.4%) had an ECOG score of 0, and 5 participants (8.1%) were smokers. A total of 36 participants (58.1%) had undergone gastrectomy before enrollment. and 30 participants (48.4%) had confirmed *Helicobacter pylori* infection. There were 49 participants (79.0%) with progressive disease (PD) after first-line chemotherapy, and 13 (21.0%) were intolerant to first-line chemotherapy. A total of 39 participants (62.9%) had cancers that were PD-L1 positive (CPS ≥ 1), and 23 were PD-L1 negative. 11 (17.7%) participants harbored TMB ≥10 mutations/Mb (TMB-H), including 3 with PD-L1 positive and 2 with MSI-high (MSI-H). In our study, no EBV-positive cases were found (Supplementary Fig. S1).

**Table 1.** Patient characteristics (*N* = 62).

Characteristics	*n* (%) or median (range)
Age, years, median (range)	62 ± 11 (95% CI: 26-86)
Gender, n	
Male	37 (59.7%)
Female	25 (40.3%)
BMI (kg/m^2^)	19.4 ± 3.4 (95% CI: 14.2-29.7)
ECOG	
0	12 (19.4%)
1	50 (80.6%)
Smoker	5 (8.1%)
Surgery	36 (58.1%)
HP (+)	30 (48.4%)
CPS ≥1	39 (62.9%)
TMB ≥10	11 (17.7%)
MSI-H	2 (3.2%)

### Efficacy

**Figure 1.**
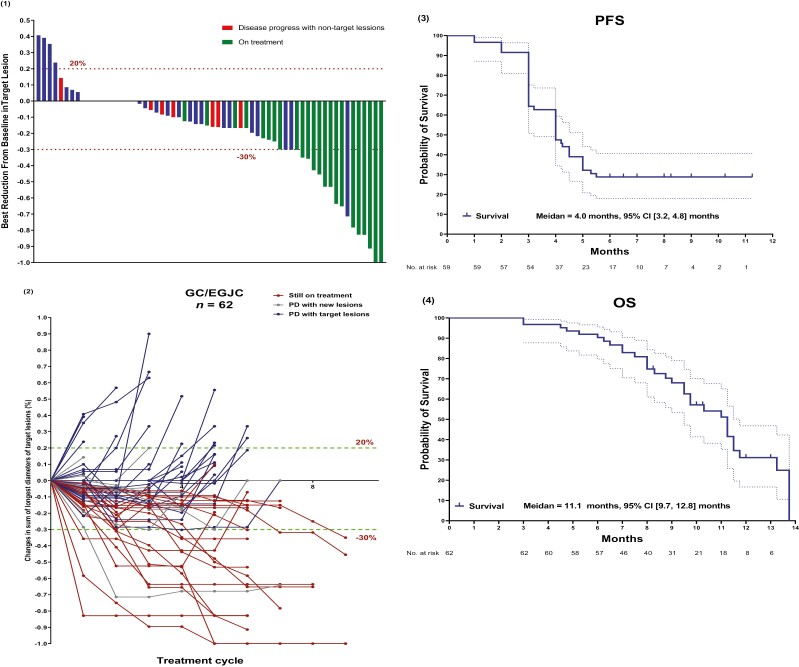
Efficacy analysis. **(1)** Waterfall plot of best percentage change from baseline in size of target tumor lesion; **(2)** Percentage change in lesion diameters over time; **(3)** Kaplan-Meier estimates of progression-free survival (PFS); **(4)** Kaplan-Meier estimates of overall survival (OS).

The primary assessment is summarized in Table 2. Analysis of the best reduction in target lesions showed that 20 participants (32.3%) achieved PR (cases example shown in Fig. 3(3) and Supplementary Fig. 2), 37 (59.7%) participants achieved stable disease (SD), and only 5 (8.1%) participants had PD as the best response (Fig. 1(1), Table 2). The ORR was 32.3%, and the disease control rate (DCR) was 91.9% (Table 2). The response duration was 8.4 months (95% CI, 3.6-10.4 months). Of the 39 participants who discontinued treatment, 7 developed new lesions (Fig. 1(1) and (2)), and 16 died of disease progression. At the time of data cutoff, 23 participants (37.1%) remained on study treatment and 23 participants (37.1%) received subsequent chemotherapy. The PFS and OS were 4.0 months (95% CI, 3.2-4.8 months) and 11.1 months (95% CI, 9.7-12.8 months), respectively (Fig. 1(3) and (4)).

**Table 2.** Primary assessment.

Primary evaluation method: Overall assessment	
Number of patients screened	68
Number of patients enrolled	62
Number of patients evaluated for toxicity	62
Number of patients evaluated for efficacy	62
Evaluation method	RECIST 1.1
Response assessment PR	n = 20
Response assessment SD	n = 37
Response assessment PD	n = 5
ORR	32.3% (20/62)
DCR	91.9% (57/62)
(Median) duration assessments PFS	8.4 months (95% CI: 3.6-10.4 months)

### Safety

Commonly seen TRAEs were fatigue (55/62, 88.7%), hypertension (44/62, 71.0%), pruritus (23/62, 37.1%), leukopenia (21/62, 33.9%), proteinuria (11/62, 17.7%), and thrombocytopenia (6/62, 9.7%). Grade 2 TRAEs were observed in 14 participants (22.6%), with 3 patients subsequently having their anlotinib dose reduced to 10 mg/day. Of the 62 participants, 7 (11.3%) experienced grade 3 TRAEs (Common Terminology Criteria for Adverse Events 5.0^32^), including 4 proteinuria cases, 2 thrombocytopenia cases, and 1 hypertension case (Table 3). Among the 7 patients with grade 3 TRAEs, dose reduction was required in 6 cases (4 patients had their anlotinib dose reduced to 10 mg/day, and 2 patients weighting less than 50 kg had their anlotinib dose reduced to 8 mg/day), and treatment cessation was required in 1 case. No treatment-related deaths occurred. No grade 4 or 5 TRAEs occurred.

**Table 3.** Adverse events.

Adverse events	Frequency	≥ Grade2	Dose adjustment	≥ Grade3	Dose adjustment
Fatigue	88.7% (55/62)	0	0	0	0
Dizziness	3.2% (2/62)	0	0	0	0
Numbness in the hands and feet	1.6% (1/62)	0	0	0	0
Hypertension	71.0% (44/62)	8	2 (anlotinib 10 mg)	1	Terminate
Pruritus	37.1% (23/62)	0	0	0	0
Thyroid dysfunction	21.0% (13/62)	0	0	0	0
Pneumonia	1.6% (1/62)	1	0	0	0
Fever	0	0	0	0	0
Leukopenia	33.9% (21/62)	1	0	0	0
Neutropenia	1.6% (1/62)	0	0	0	0
Thrombocytopenia	9.7% (6/62)	0	0	2	2 (1 delayed anlotinib to 10 mg and 1 delayed anlotinib dosage to 8 mg)
Proteinuria	17.7% (11/62)	4	1 (anlotinib 10 mg)	4	4 (3 delayed anlotinib dose to 10 mg and 1 delayed anlotinib dose to 8 mg)
Nausea	12.9% (8/62)	0	0	0	0
Abdominal distention	6.5% (4/62)	0	0	0	0
Abdominal pain	4.8% (3/62)	0	0	0	0
Diarrhea	12.9% (8/62)	0	0	0	0
Vomiting	8.1% (5/62)	0	0	0	0
Constipation	6.5% (4/62)	0	0	0	0
AST increased	14.5% (9/62)	2	0	0	0
ALT increased	17.7% (11/62)	2	0	0	0
Blood bilirubin increased	6.5% (4/62)	0	0	0	0
Amylase increase	12.9% (8/62)	0	0	0	0

Adverse events were classified according to the National Cancer Institute Common Terminology Criteria (CTCAE) version 5.0.

Abbreviations: AST, aspartate aminotransferase; ALT, alanine aminotransferase.

### PD-L1 Expression

**Figure 2.**
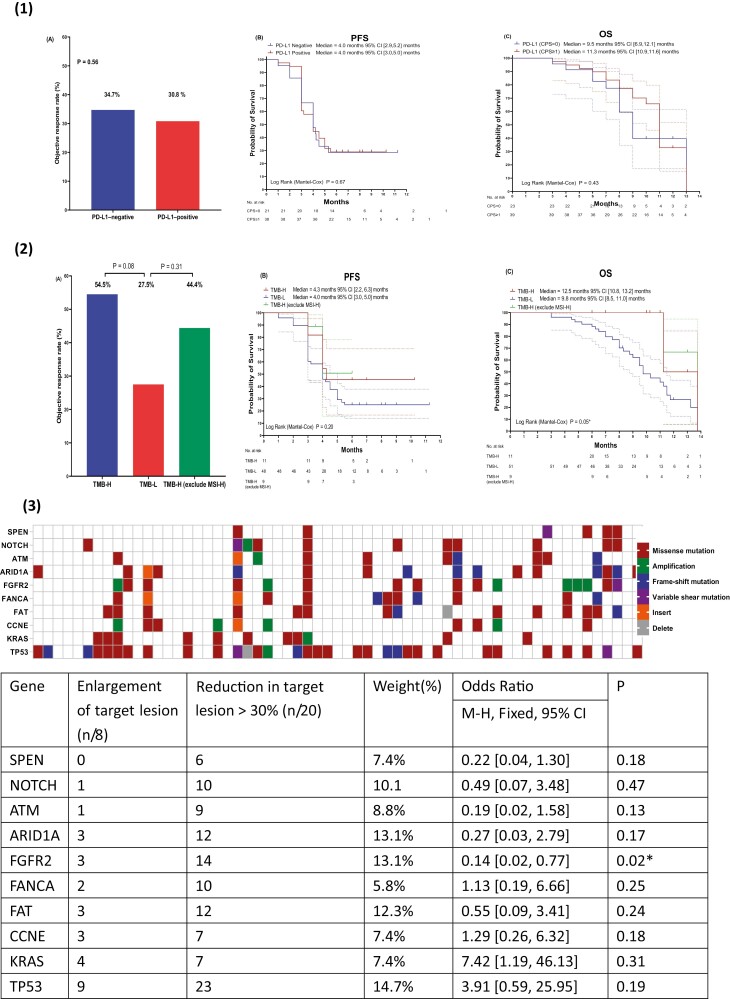

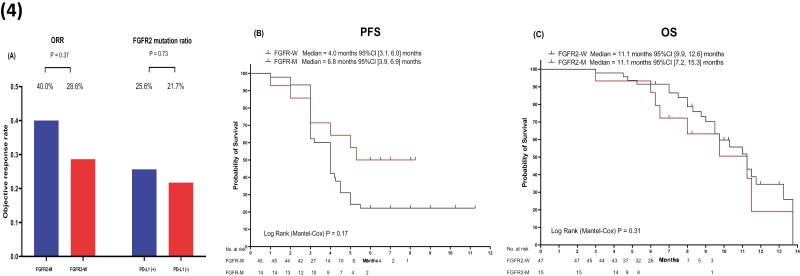
**(1)** ORR **(A)**, PFS **(B)** and OS **(C)** of PD-L1 negative or positive patients; **(2)** ORR **(A)**, PFS **(B)** and OS **(C)** of TMB-H, TMB-L and TMB-H (excluding MSI-H); **(3)** Gene mutation analysis of all the enrolled patients; **(4)** The ORR of FGFR2-mutated patients, and the FGFR2 mutation ratio in PD-L1 positive or TMB-H groups **(A)**, PFS **(B)** and OS **(C)** of FGFR2-mutated patients.

Positive PD-L1 expression was found in 39 of 62 participants (CPS ≥ 1) (Table 1). The ORR was 34.7% and 30.8% in the PD-L1-negative group and PD-L1-positive group (*P = .*56), respectively (Fig. 2(1)A). The PFS curves of the PD-L1 positive and negative groups were tightly intertwined (PD-L1 negative group: 4.0 months, 95% CI, 2.9-5.2 months; PD-L1 positive group: 4.0 months, 95% CI, 3.0-5.0 months; *P = .*67) (Fig. 2(1)B). The PD-L1 positive and negative groups showed OS of 11.3 months (95% CI, 10.9-11.6 months) and 9.5 months (95% CI, 6.9-12.1 months), respectively (*P = .*43) (Fig. 2(1)C).

### TMB and MSI Status

Eleven of the enrolled participants had a high TMB of ≥10 mutations/Mb (Table 1). There were 3 patients who were both PD-L1 positive and had TMB-H. Patients with TMB-H and TMB-L had an ORR of 54.5% and 27.5% (*P = .*08, Fig. 2(2)A), respectively, in the combination treatment. Survival analysis indicated the PFS of the TMB-H and -L groups were 4.3 months (95% CI 2.2-6.3) and 4.0 months (95% CI 3.0-5.0 months) (*P = .*20; Fig. 2(2)B), respectively. The TMB-H group exhibited a much longer OS than TMB-L group (TMB-H group: 12.5 months, 95% CI 10.8-13.2 months; TMB-L: 9.8 months, 95% CI 8.5-11.0 months; *P = .*05; Fig. 2(2)C). There were 2 TMB-H patients who were also had MSI-H tumors (Table 1), both of whom achieved PR as the best response. When the 2 MSI patients were excluded, the ORR of the TMB-H group was 44.4%, which was still tend to numerically higher than the TMB-L group (*P = .*31, Fig. 2(2)A). Due to the limited number of cases survival analysis could not been defined (Fig. 2(2)B and C).

### FGFR2

We integrated tumor samples to identify molecular features that predicted sensitivity or primary resistance to this experimental combination. The NGS analysis indicated that there were 15 participants with *FGFR2* mutations (Fig. 2(3)). The *FGFR2* mutation significantly benefited target lesion reduction (odds ratio [OR] = 0.22; *P* = .02; Fig. 2(3)). Patients with *FGFR2* mutation (*FGFR2*-M) showed a numerically higher ORR and longer PFS than those patients with wild-type (FGFR2-W) (ORR: 40% vs 28.6%, *P = .*37; PFS: *FGFR2-*M: 6.8 months 95% CI, 3.9-6.9, *FGFR2*-W: 4.0 months 95% CI, 3.1-6.0 months, *P = .*17) (Fig. 2(4) A and B). However, OS was similar between the 2 groups (*FGFR2-*M: 11.1 months 95% CI, 7.2-15.3 months, *FGFR2*-W: 11.1 months 95% CI, 9.9-12.6, *P = .*31) (Fig. 2(4) C).

### Other Biomarkers and Subgroup Analysis

Other patient characteristics, including age, gender, surgery, and *H. pylori* infection, had no statistically significant association with clinical efficacy (Supplementary Fig. S3(1)-(8)). Although the number of participants in this study was limited, there tended to be a better response for patients with mutated *SPEN*, *NOTCH*, *ATM*, *ARID1A*, and *FAT* genes than the wild-type ones, while *CCNE*, *KRAS*, and *TP53* mutations appeared to be negatively correlated with response to the combination regimen (Fig. 2(3)).

### TME Assessment

**Figure 3.**
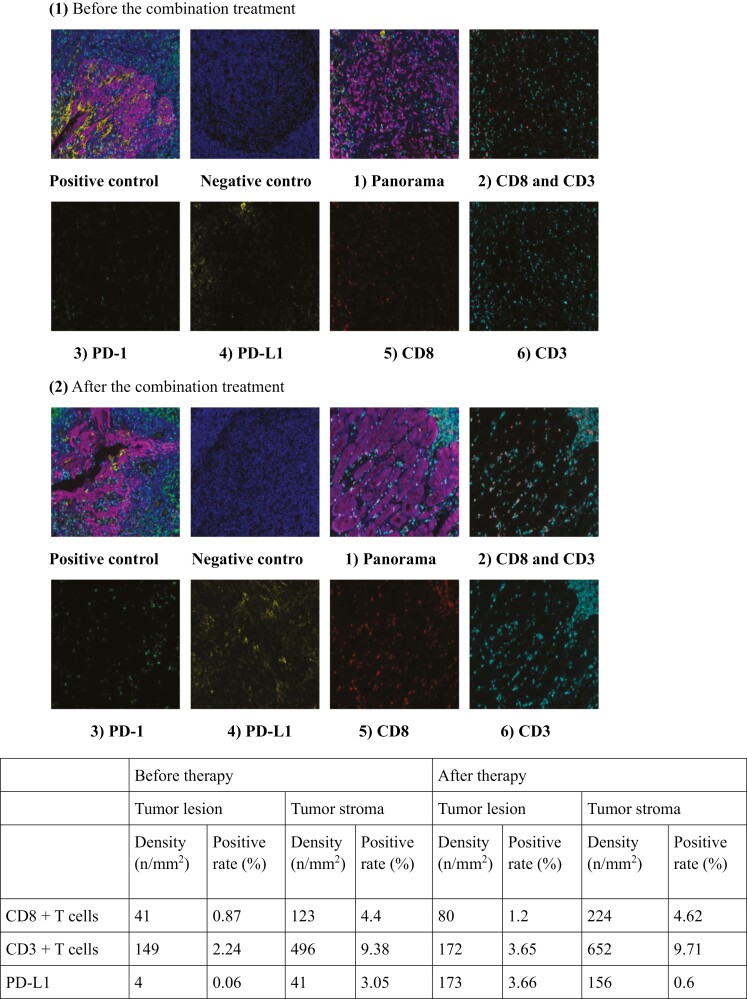

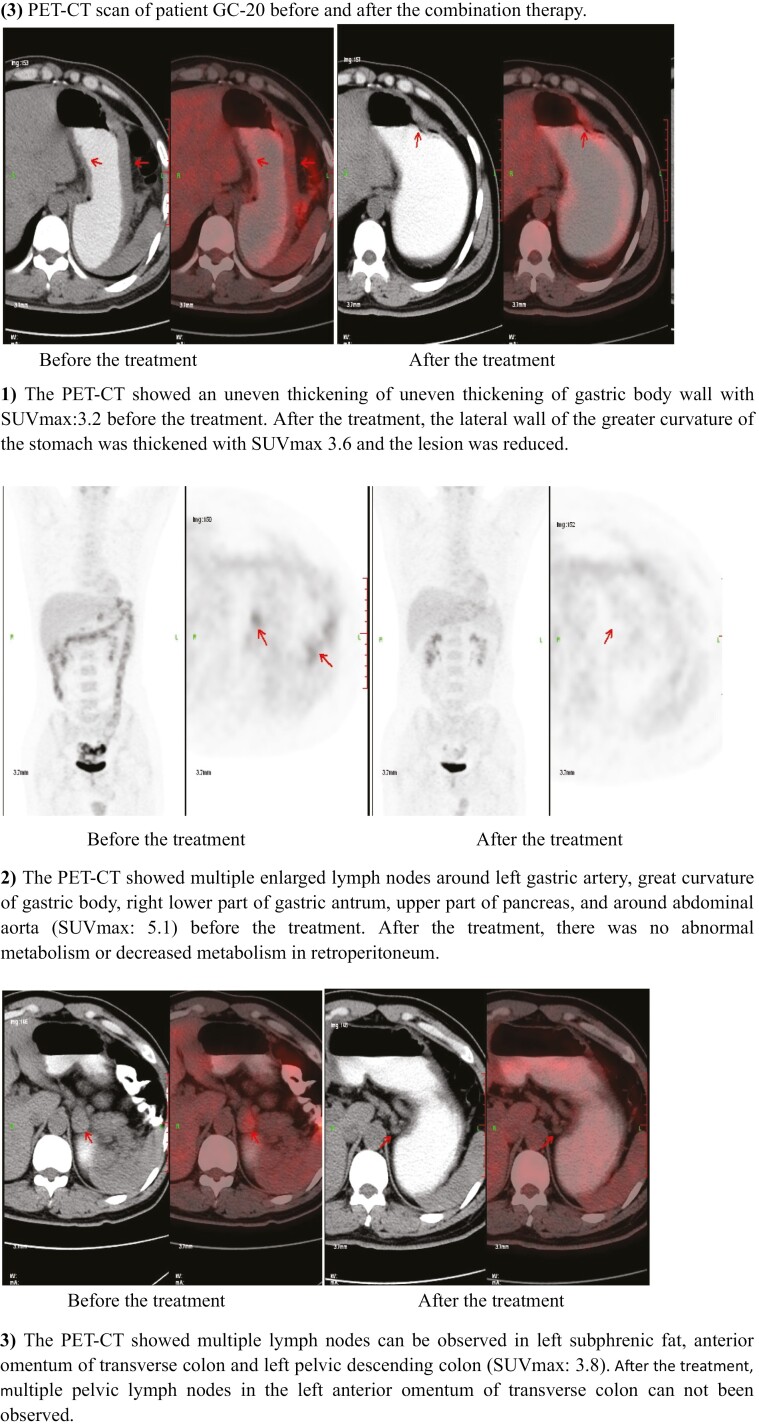
Tumor microenvironment analysis of patient GC-20 who was underwent surgery after 8 courses of combination treatment: **(1)** The immune microenvironment of the tumor before combination treatment: **1)** the immune microenvironment panorama, **2)** expression of CD3 and CD8, **3)** expression of PD-1, **4)** expression of PD-L1, **5)** expression of CD8, **6)** expression of CD3; **(2)** The immune microenvironment of the tumor after combination treatment: **1)** the immune microenvironment panorama, **2)** expression of CD3 and CD8, **3)** expression of PD-1, **4)** expression of PD-L1, **5)** expression of CD8, **6)** expression of CD3 (CD8: red; CD3: cyan; PD-L1: yellow; PD-1: green; panCK: purple; DAPI: blue); **(3)** PET-CT scan of patient GC-20 before and after the combination therapy: **1)** the PET-CT showed an uneven thickening of uneven thickening of the gastric body wall with (SUVmax:3.2) before treatment. After treatment, the lateral wall of the greater curvature of the stomach was thickened (SUVmax 3.6), and the lesion was red uced; **2)** the PET-CT showed multiple enlarged lymph nodes around left of the gastric artery, great curvature of the gastric body, right lower part of the gastric antrum, upper part of the pancreas, and around the abdominal aorta (SUVmax: 5.1) before the treatment. After the treatment, there was no abnormal metabolism or decreased metabolism in the retroperitoneum; **3)** the PET-CT showed multiple lymph nodes could be observed in left subphrenic fat, the anterior omentum of the transverse colon and the left pelvic descending colon (SUVmax: 3.8). After treatment, multiple pelvic lymph nodes in the left anterior omentum of transverse colon could not been observed.

To observe changes in the microenvironment during treatment with anlotinib and toripalimab, T-cell infiltration in the selected patient tumor tissue was compared before and after the treatment (Fig. 3). As shown in Fig. 3(1) and (2), the infiltration of CD3^+^ T and CD8^+^ T cells increased significantly after therapy. As shown in Fig. 3, combination treatment increased the densities and positivity rates of CD8^+^ T cells, CD3^+^ T cells, and PD-L1 expression in tumor lesions. However in the tumor stroma, the positivity rates of CD8^+^ T cells, CD3^+^ T cells, and PD-L1 expression did not increase. These findings indicated that the combined treatment of anlotinib and toripalimab promoted an immune-supportive environment in tumor lesions.

### Organoids Studies

**Figure 4.**
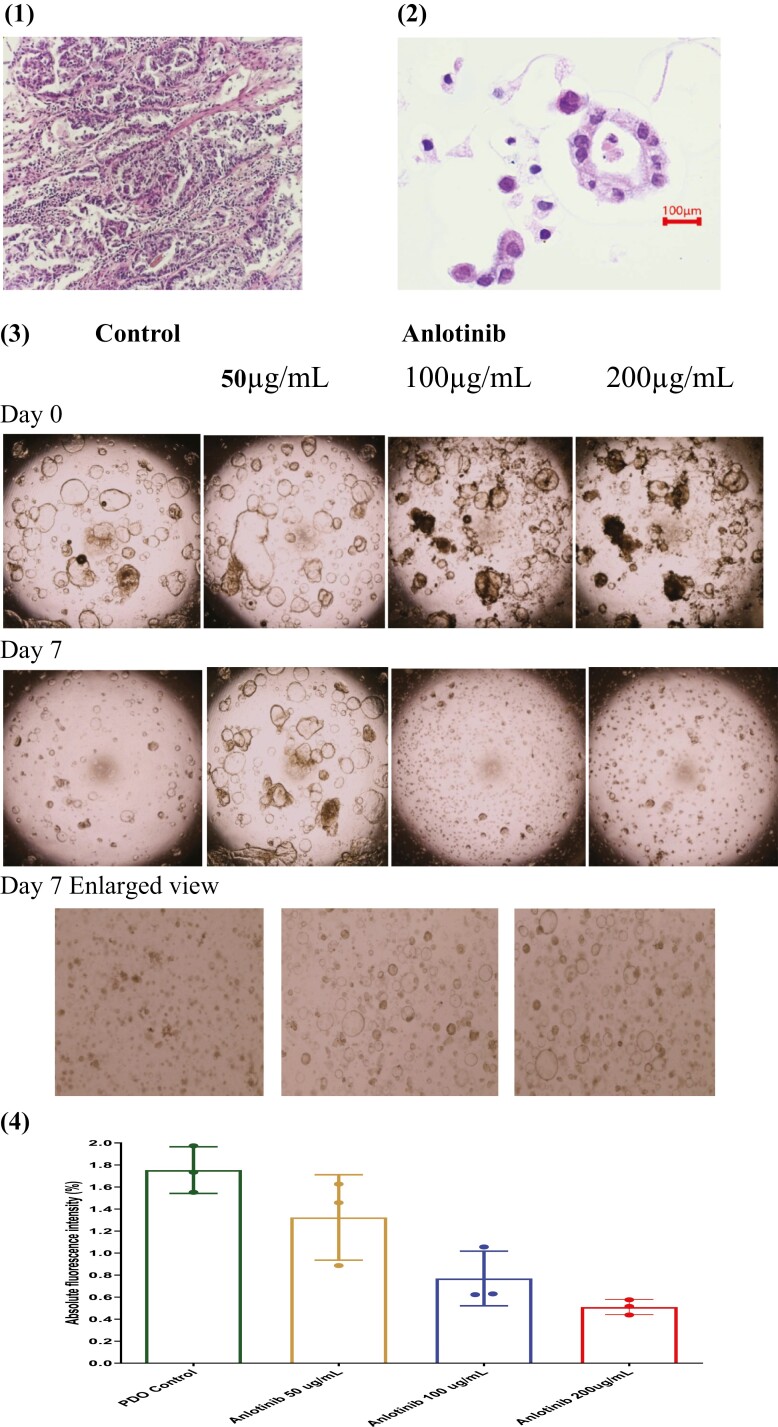

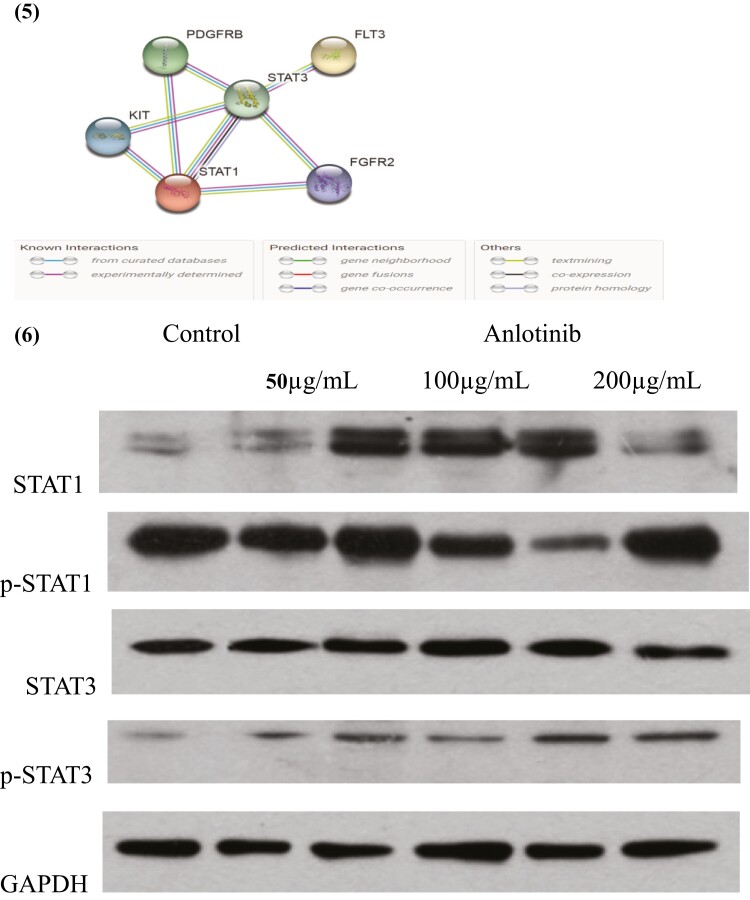
PDO studies. **(1)** HE staining of the patients tumor tissue; **(2)** HE staining of the patient-derived gastric cancer organoid (PDO); **(3)** the direct efficacy of anlotinib on the PDOs; **(4)** viability assay of the different treatment tumor cells from the PDOs cultivated for 7 days; **(5)** STRING analysis to study the potential mechanism; **(6)** western-blot analysis of the expression of STAT1 and STAT3 after anlotinib treatment.

We cultured PDOs to assess the regulatory role of anlotinib in the immune microenvironment. The pathological characteristics of the PDOs and the biopsy sample are shown in Fig. 4(1) and (2), respectively. As shown in Fig. 4(3), the cell spheres were treated with PBS and anlotinib (50 µg/mL, 100 µg/mL, or 200 µg/mL), respectively. After 7 days of treatment, tumor cell spheres enlarged in the control group, while all the anlotinib treatment groups showed shrinkage of the cell spheres (Fig. 4(3)). As shown in Fig. 4(4), compared to the control group, anlotinib dose-dependently reduced the viability of the tumor cells. An interaction network for the proteins of *FGFR2*, *PDGFRB*, *KIT*, and *FLT3*, which are targets of the anlotinib and the *JAK/STAT* pathway, was generated by the STRING database (version 11.0).^32^ As shown in Fig. 4(5), the anlotinib therapy targets, including *FGFR2*, *PDGFRB*, *FLT3*, and *KIT*, exhibited co-occurrences and neighbored he *STATS* signaling pathway. To study the potential mechanism of anlotinib on immune activation, western blotting was performed to quantitatively evaluate the expression of *STAT1*, *STAT3*, *p-STAT*, and *p-STAT3* in anlotinib treatment. As shown in Fig. 4(6), as expected, the expression of *p-STAT1* and *p-STAT3* amounts increased with the administration of anlotinib, indicating that anlotinib could potentially promote the response to immunotherapy.

## Discussion

Herein, we first reported for the first time safety and efficacy of combined anlotinib with toripalimab as second-line treatment in advanced, relapsed Chinese GC/GEJC patients. This study showed that this new regimen had a promising safety profile. The incidence of grade 3 or above TRAEs was much lower than that reported in a previous study of anti-PD-1 antibody combined with chemotherapy^33^ or other TKIs.^8,10,11^ The primary efficacy endpoint was reached, with 20 patients achieving PR (ORR 32.3%, 95% CI, 26.6-38.5%), which was a promising result for GC/GEJC.^3^ The PFS (4.0 months, 95% CI, 2.5-5.5 months) and OS (11.1 months, 95% CI, 9.7-12.8 months) were both longer than those reported in previous studies of anti-PD-1 monotherapy or apatinib combined with a PD-1 inhibitor in second-line settings.^3,11^ These results jointly suggested that the combination of anlotinib and toripalimab had promising efficacy for GC/GEJC patients as second-line treatment.

The expression of PD-L1 has been reported to be correlated with enhanced clinical response to PD-1 antibody monotherapy or combination therapy with chemotherapy. However, in our study the differences in ORR and PFS in the PD-L1 positive group and negative group were not significant. The small number of participants may have limited the assessment of biomarkers. Conversely, the addition of anlotinib in our study may have enhanced the susceptibility of the PD-L1 negative or low-expression patients to PD-1 antibody. Antiangiogenic agents have been reported to modulate the TME and improve immunotherapy.^8,9,12,14^ In the study by Fukuoka et al, the antiangiogenic agents had the potential to overcome resistance of anti-PD-1 therapy. The combination of low-dose regorafenib plus nivolumab exhibited synergistic effect, sensitizing immunotherapy and reducing toxicity in gastric cancer.^8^ In a patient treated with toripalimab plus anlotinib, the amount of infiltration CD8^+^ T cells and CD3^+^ T cells significantly increased in tumor tissue, indicating the combination therapy promoted an immune-supportive environment. In addition, the western blot results showed that levels of phosphorylated *STAT1/STAT3* was elevated by anlotinib. We know that *STAT1/STAT3* phosphorylation is a key event in PD-L1 expression and increases the amount of inflammatory-promoting T cell infiltration in tumor tissue.^12^ Base on these results, we speculated that anlotinib was conducive to developing an immune-supportive microenvironment and could potentially increasing the expression of PD-L1. In addition, it was possible that anlotinib could change “cold” tumor cells into “hot” cells and potentially expand the treatment window of anti-PD-L1 antibodies.

The TMB has been emerged as a biomarker for PD-1 antibody treatment in diverse tumor types.^34-36^ According to the KEYNOTE-158 trial, the FDA approved the use of pembrolizumab for advanced solid tumors with TMB ≥ 10 mutations/Mb that progressed with prior therapy, making it the second tumor-agnostic approval of immune checkpoint inhibitor (ICI) therapy.^37^ However in GC, the predictive effect of TMB has not yet been demonstrated. A retrospective study of advanced esophagogastric cancer by Greally et al found no association between TMB and response to ICIs, especially when MSI tumors were excluded.^38^ Another study by Wang et al, identified that Chinese chemo-refractory GC patients with TMB ≥ 12 experienced higher ORR and prolonged OS with toripalimab monotherapy than those with TMB-L.^22^ Herein, at the cutoff point of 10 mutations/Mb, patients with TMB-H also showed significantly better OS than those with TMB-L. Due to the limited number of cases in this study, and because the study participants included solely Chinese patients, only a potential tendency can be speculated from the results.

We used NGS analysis to explore novel potential prognostic biomarkers. The *FGFR2* gene, which was the target of anlotinib, was potentially associated with the prognosis of the combination therapy. It has been reported that *FGFR2* mutations, which may lead to an overexpression of *FGFR2*, could be a key driver in the upregulation of PD-L1 expression.^38^ More patient samples should be collected before and during therapy in future studies to identify new biomarkers for the combination treatment.

### Limitations

Some limitations of this study should be noted. First, the number of participants was relatively low, and because this was an exploratory trial, the small sample size might have been a critical factor affecting the outcome. A future clinical trial with a larger population is thus warranted. In addition, the observation period was short. This was a single-arm study, and a randomized, controlled phase III clinical trial should be designed to verify the findings. Second, we only explored efficacy in the Chinese population. The biological characteristics of gastric cancers from different populations vary considerably.^39^ Caution is needed before applying the findings of our study to other populations, such as European and Japanese. Third, all participants enrolled in our study had EBV-negative status. According to our results, anlotinib could enhance the susceptibility of patients to PD-1 inhibitors. Further, the novel regimen of anlotinib plus toripalimab is benefited to GC/GEJC patients regardless of PD-L1 status. There is a need for a future clinical trials of this novel combination with a better-defined patient population which includes the PD-L1 negative patients.

## Conclusions

In this study, anlotinib-toripalimab combination therapy showed adequate tolerance and promising efficacy in patients with advanced GC/GEJC in this study. Based on our results, anlotinib could enhance the susceptibility of patients to PD-1 inhibitors. The addition of anlotinib to toripalimab therapy potentially regulated TME and increased PD-L1 expression in tumor lesions, expanding the treatment window of toripalimab and improving the benefit of second-line therapy in GC/GEJC. The TMB-H group showed a significantly longer OS than the TMB-L group. Moreover, *FGFR2* mutation may be a potential biomarker of therapeutic efficacy. A future clinical trial should be designed to verify these results.

## Supplementary Material

oyac136_suppl_Supplementary_Figure_S1

oyac136_suppl_Supplementary_Figure_S2

oyac136_suppl_Supplementary_Figure_S3

oyac136_suppl_Supplementary_Tables

## Data Availability

The data underlying this article will be shared on reasonable request to the corresponding author.
