# Supplementary material for: Anlotinib Combined with Toripalimab as Second-Line Therapy for Advanced, Relapsed Gastric or Gastroesophageal Junction Carcinoma
Source: Oncologist. 2022 Jul 20;27(11):e856–69. doi: 10.1093/oncolo/oyac136 (PMC9632317; doi:10.1093/oncolo/oyac136)
Supplement: oyac136_suppl_Supplementary_Figure_S1 [file oyac136_suppl_supplementary_figure_s1.docx]

**
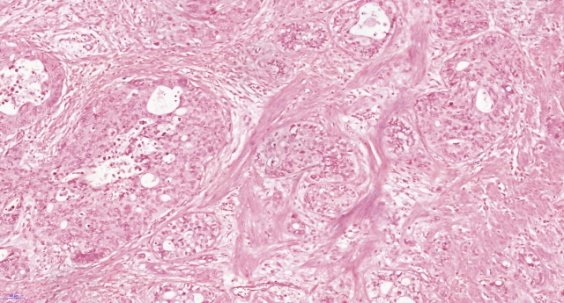

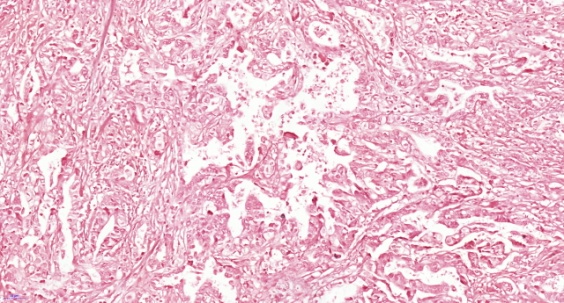
**

**G01 G02**

**
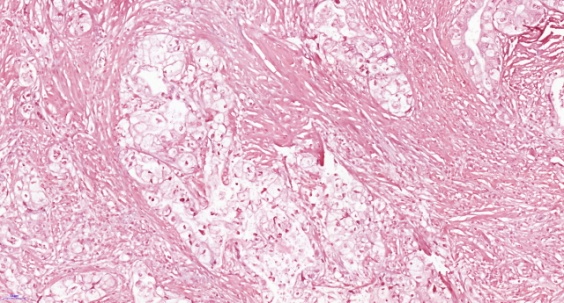

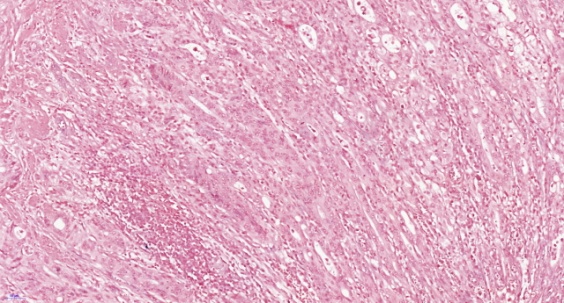
**

**G03 G04**

**
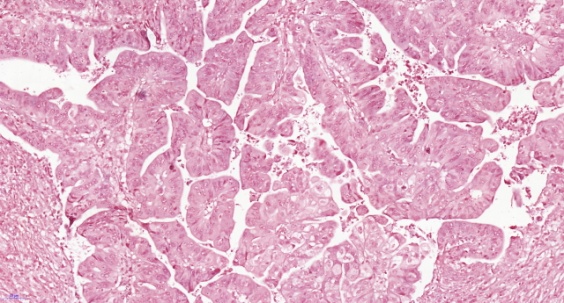

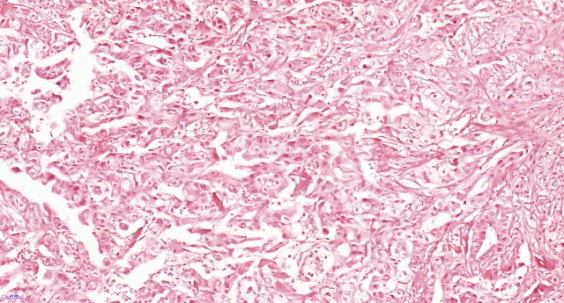
**

**G05 G06**

**
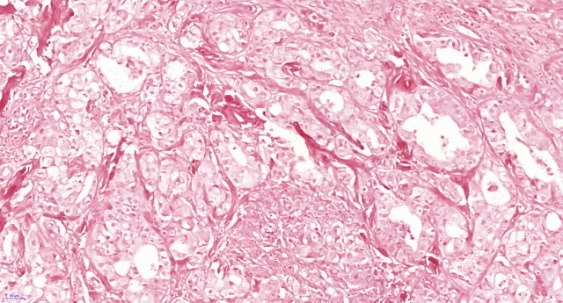

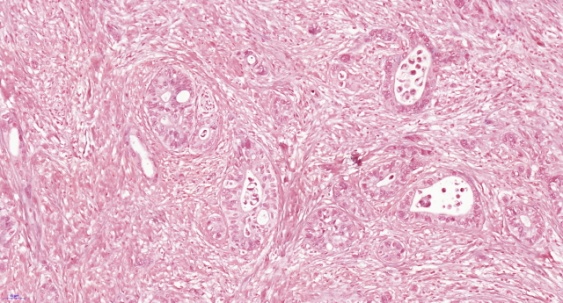
**

**G07 G08**

**
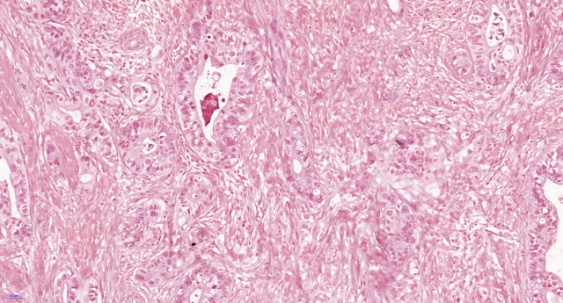

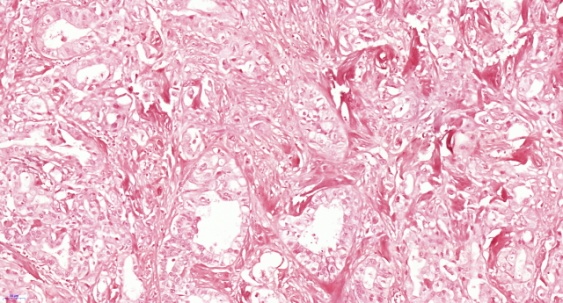
**

**G09 G10**

**
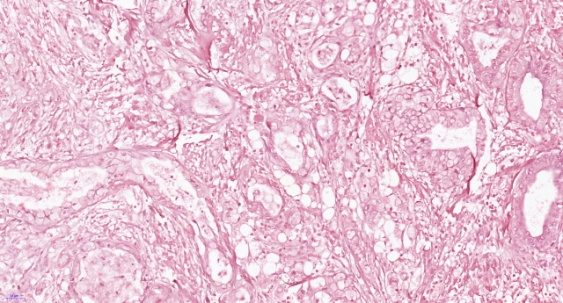

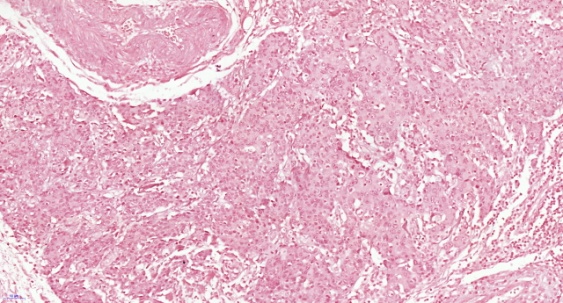
**

**G11 G12**

**
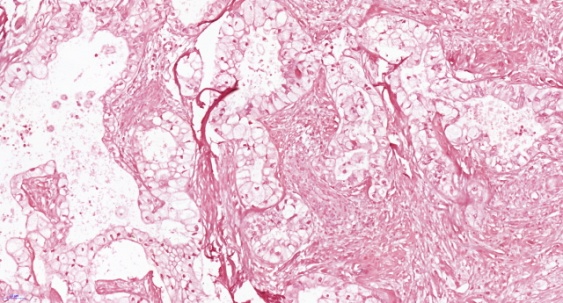

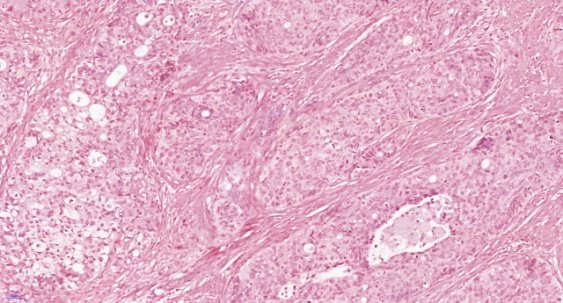
**

**G13 G14**

**
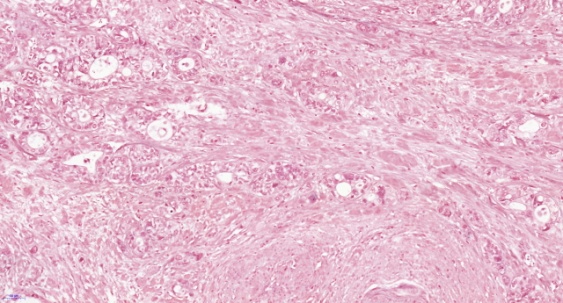

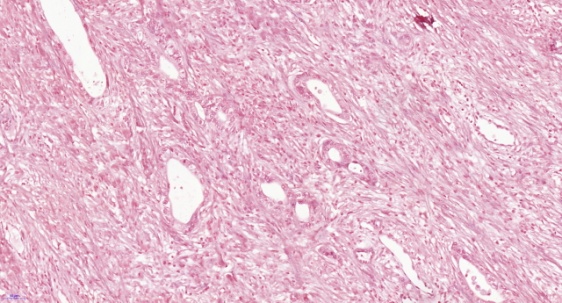
**

**G15 G16**

**
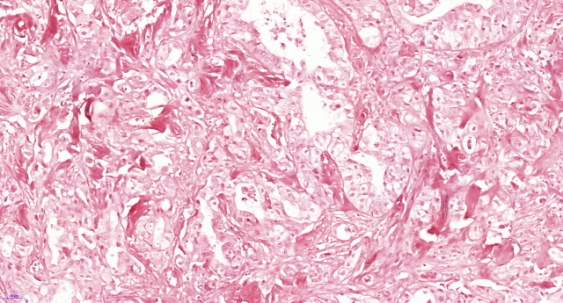

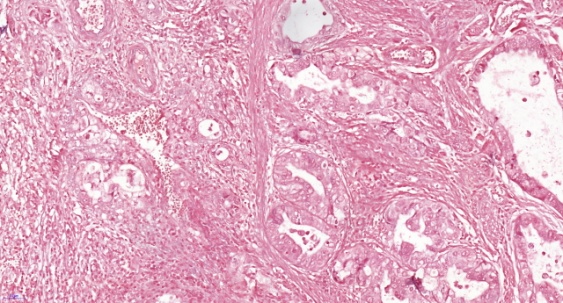
**

**G17 G18**

**
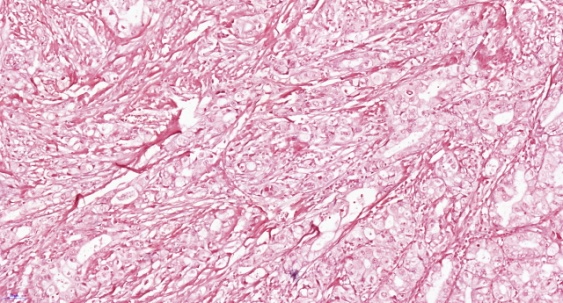

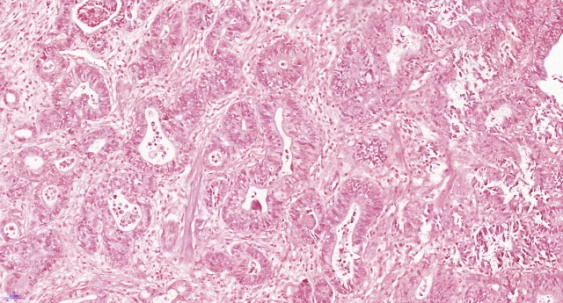
**

**G19 G20**

**
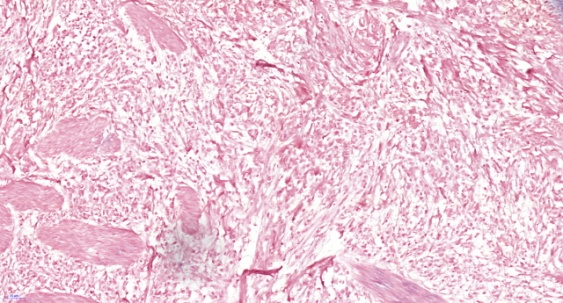

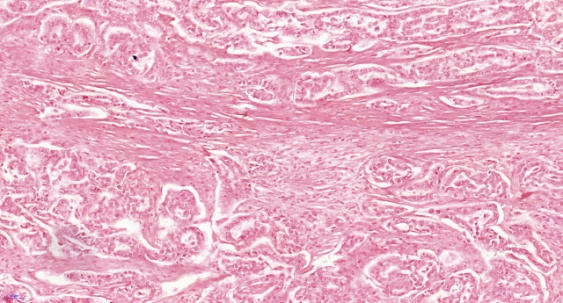
**

**G21 G22**

**
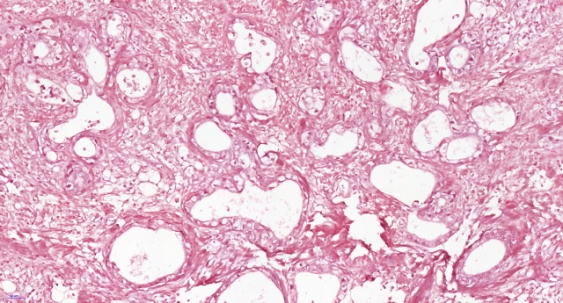

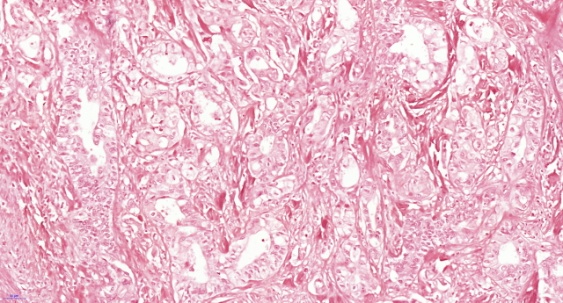
**

**G23 G24**

**
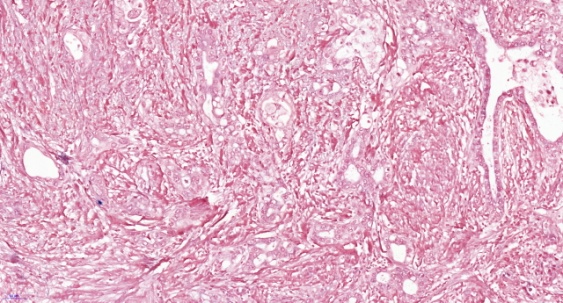

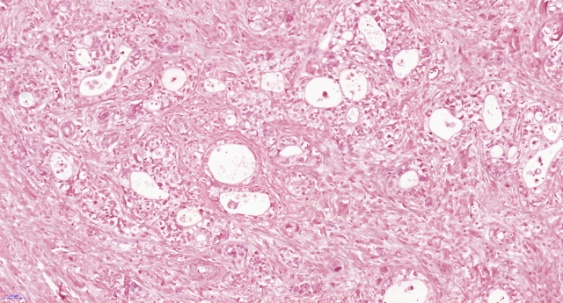
**

**G25 G26**

**
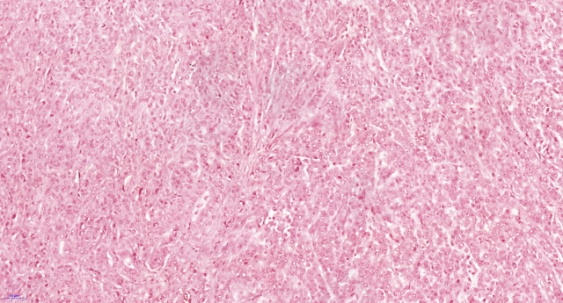

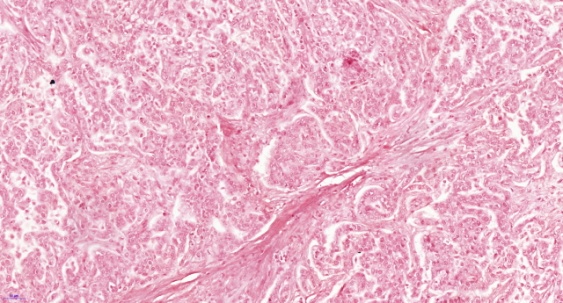
**

**G27 G28**

**
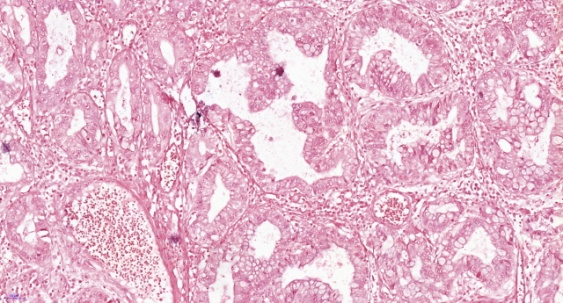

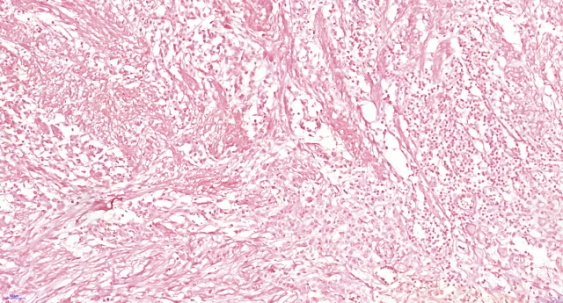
**

**G29 G30**

**
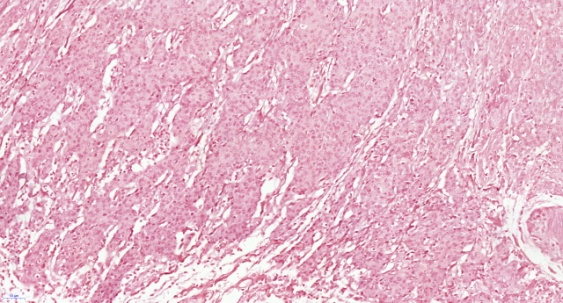

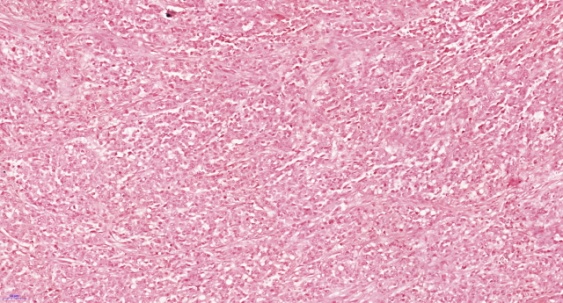
**

**G31 G32**

**
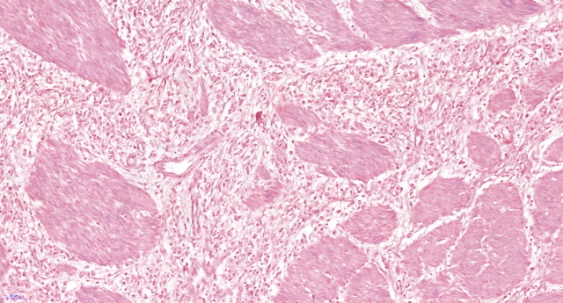

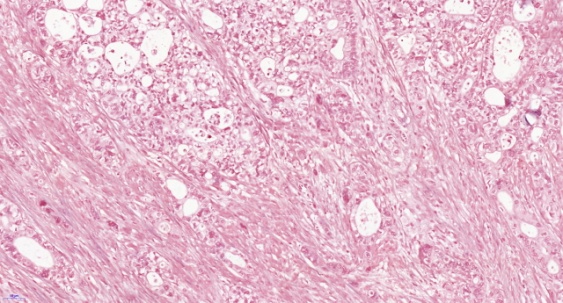
**

**G33 G34**

**
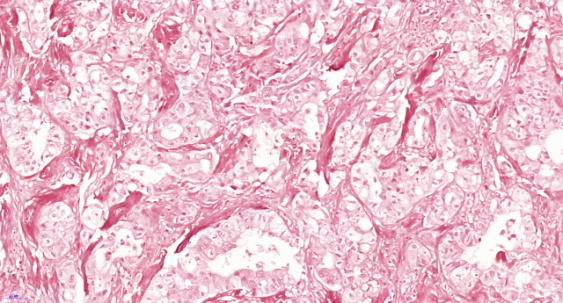

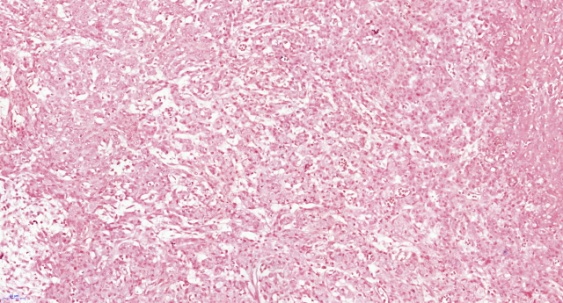
**

**G35 G36**

**
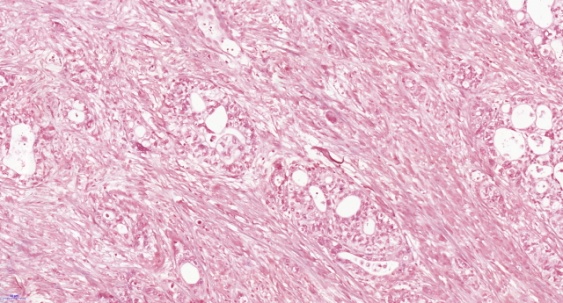

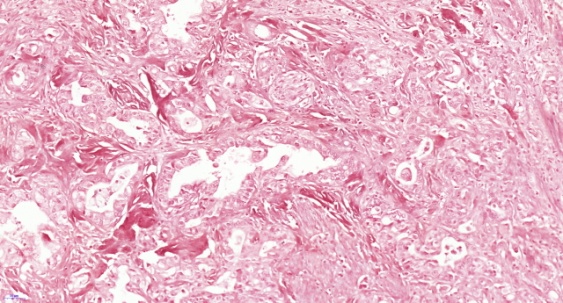
**

**G37 G38**

**
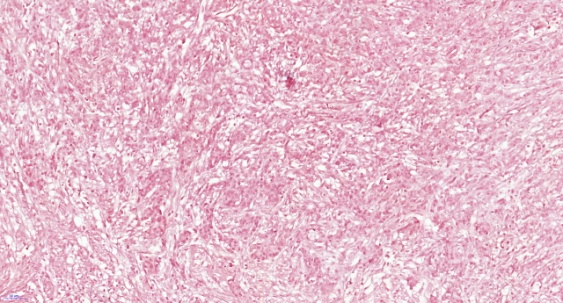

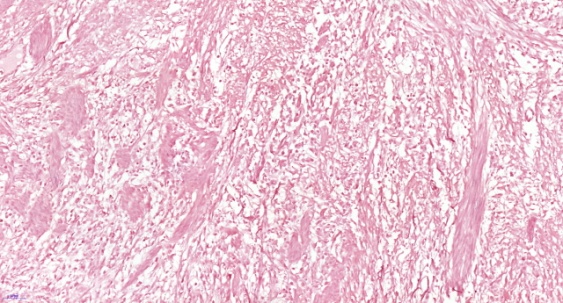
**

**G39 G40**

**
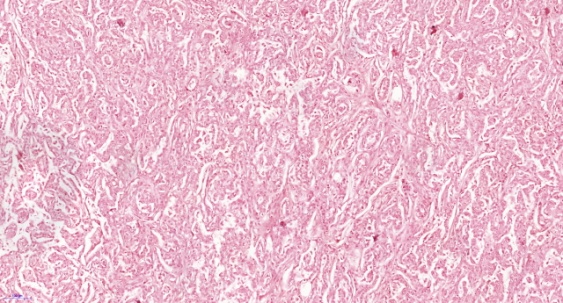

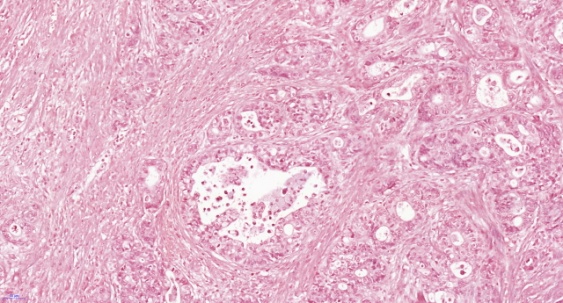
**

**G41 G42**

**
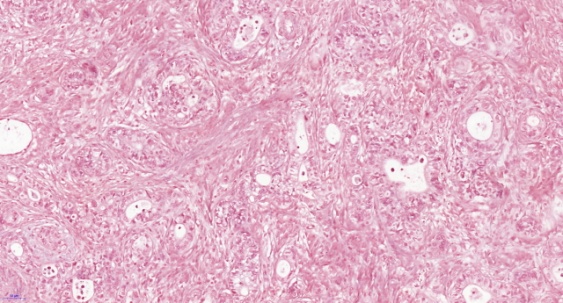

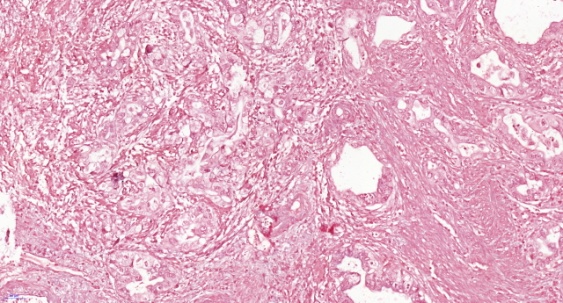
**

**G43 G44**

**
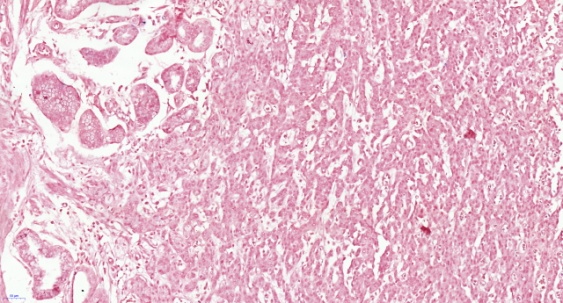

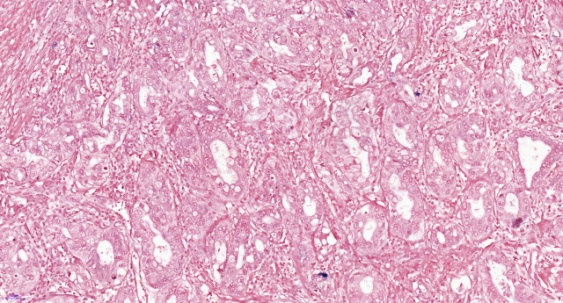
**

**G45 G46**

**
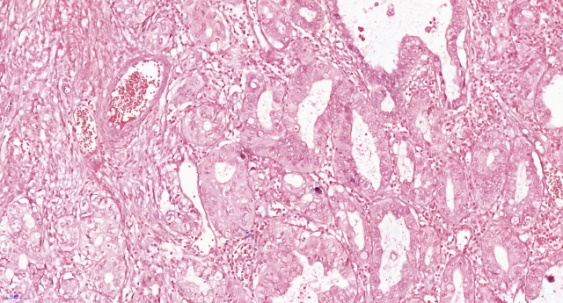

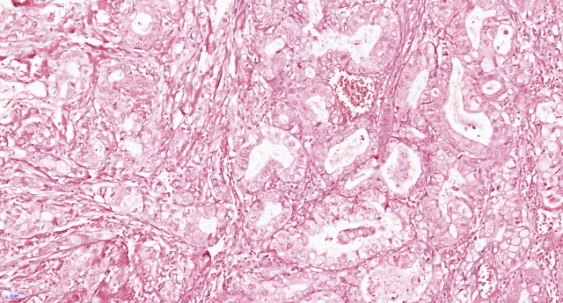
**

**G47 G48**

**
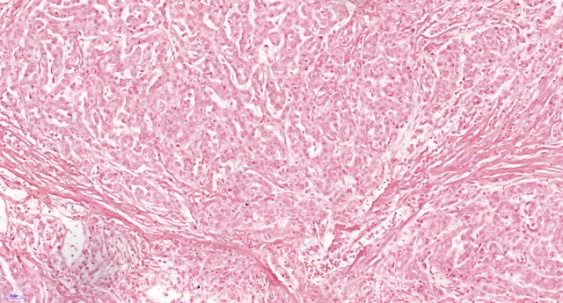

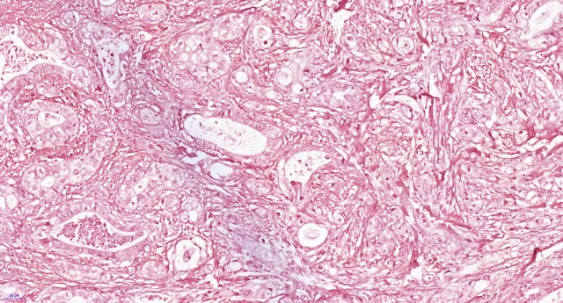
**

**G49 G50**

**
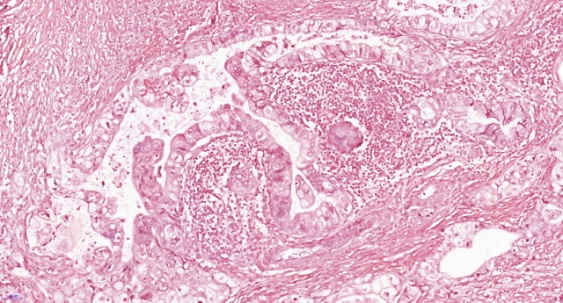

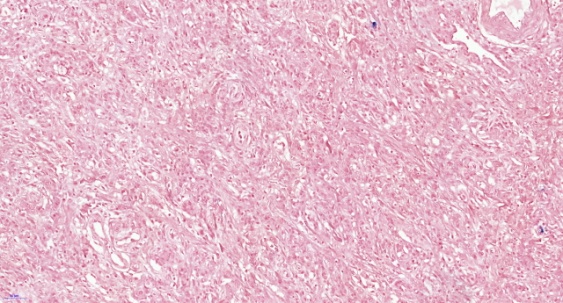
**

**G51 G52**

**
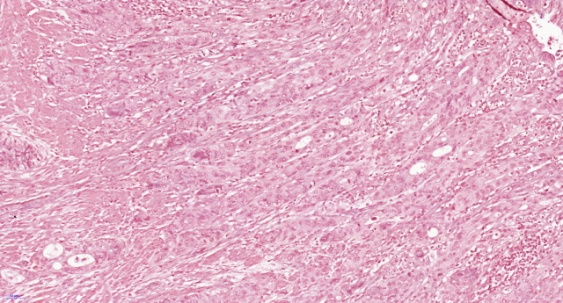

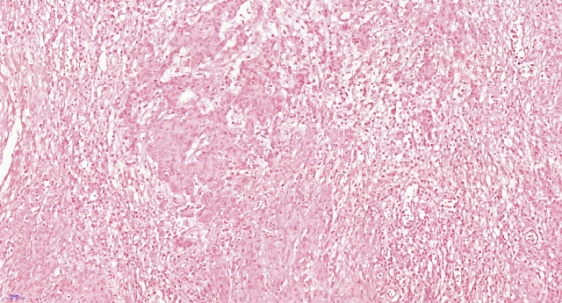
**

**G53 G54**

**
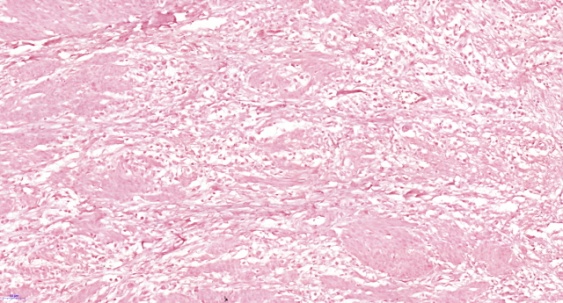

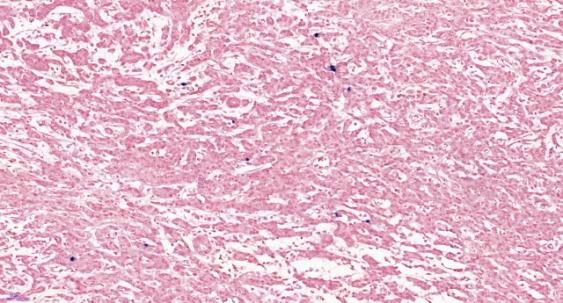
**

**G55 G56**

**
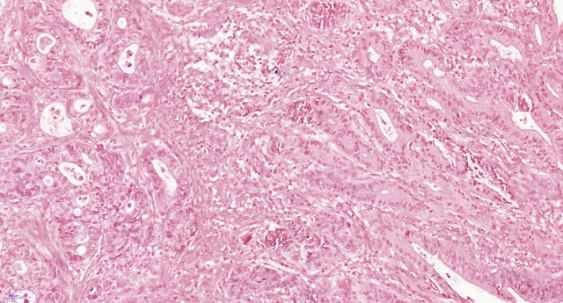

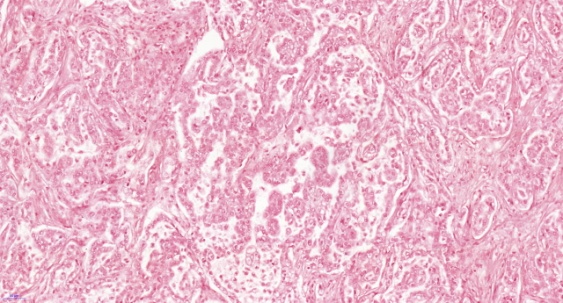
**

**G57 G58**

**
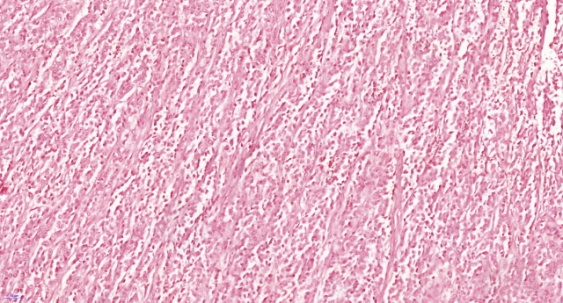

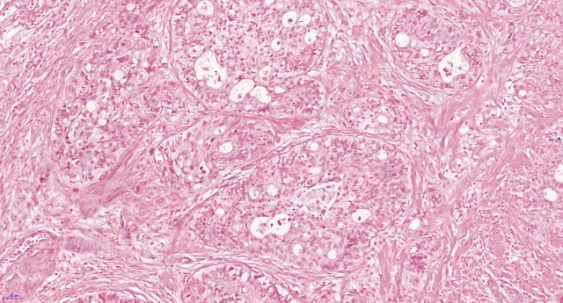
**

**G59 G60**

**
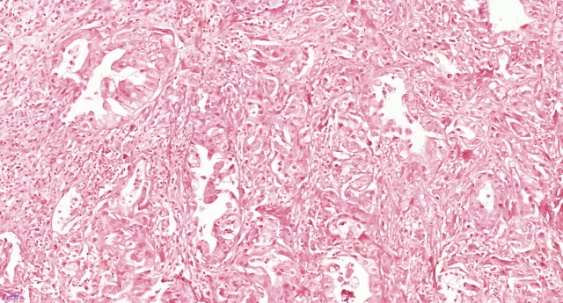

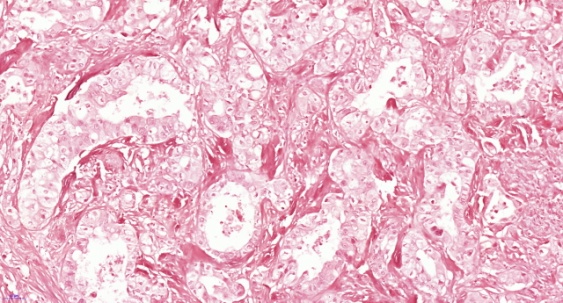
**

**G61 G62**

**Supplementary Figure 1.** The EBV status of all the enrolled patients.
