# Supplementary material for: Anlotinib Combined with Toripalimab as Second-Line Therapy for Advanced, Relapsed Gastric or Gastroesophageal Junction Carcinoma
Source: Oncologist. 2022 Jul 20;27(11):e856–69. doi: 10.1093/oncolo/oyac136 (PMC9632317; doi:10.1093/oncolo/oyac136)
Supplement: oyac136_suppl_Supplementary_Figure_S2 [file oyac136_suppl_supplementary_figure_s2.docx]

**(1)** Before the combination treatment **(2)** Surgery samples showed PCR


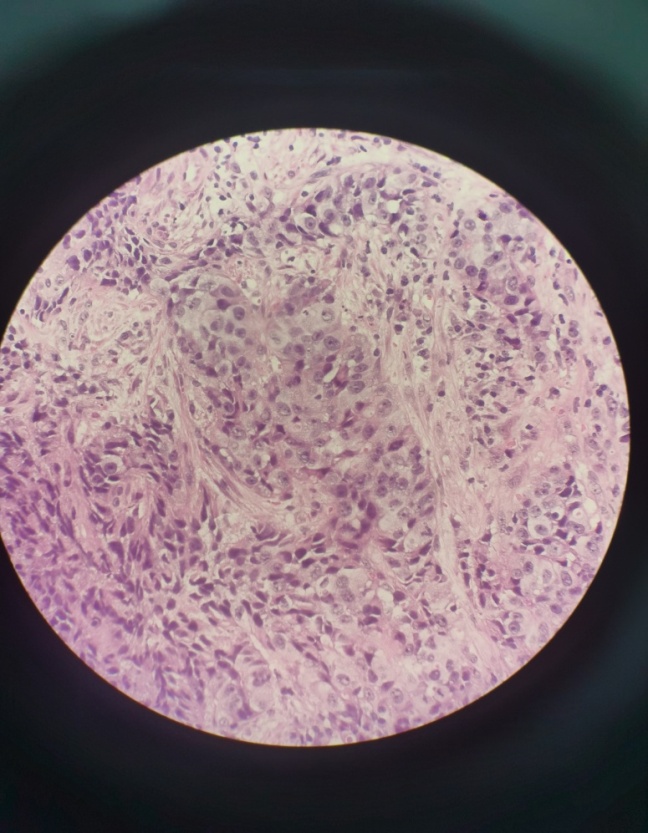

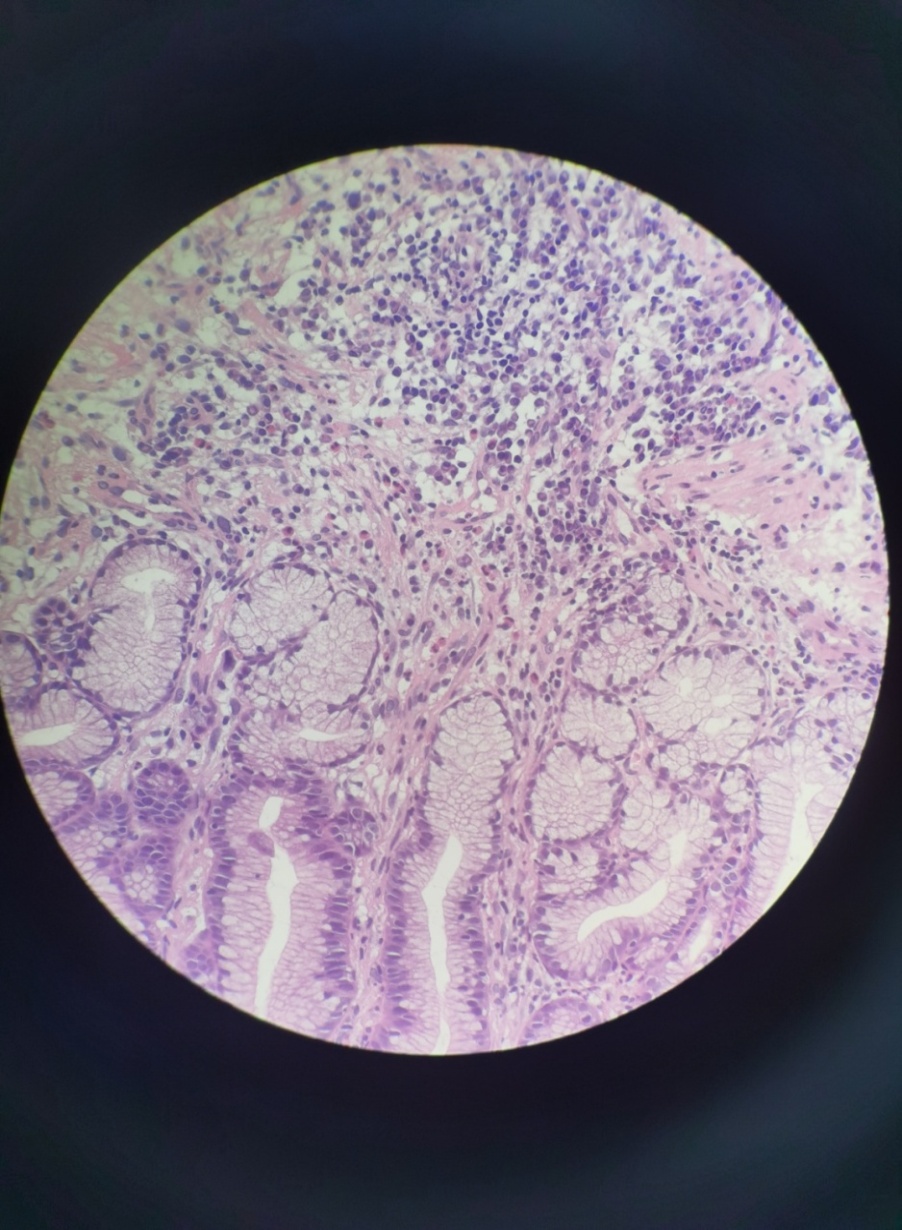


**(3)** PET-CT scan after and before the combination treatment.


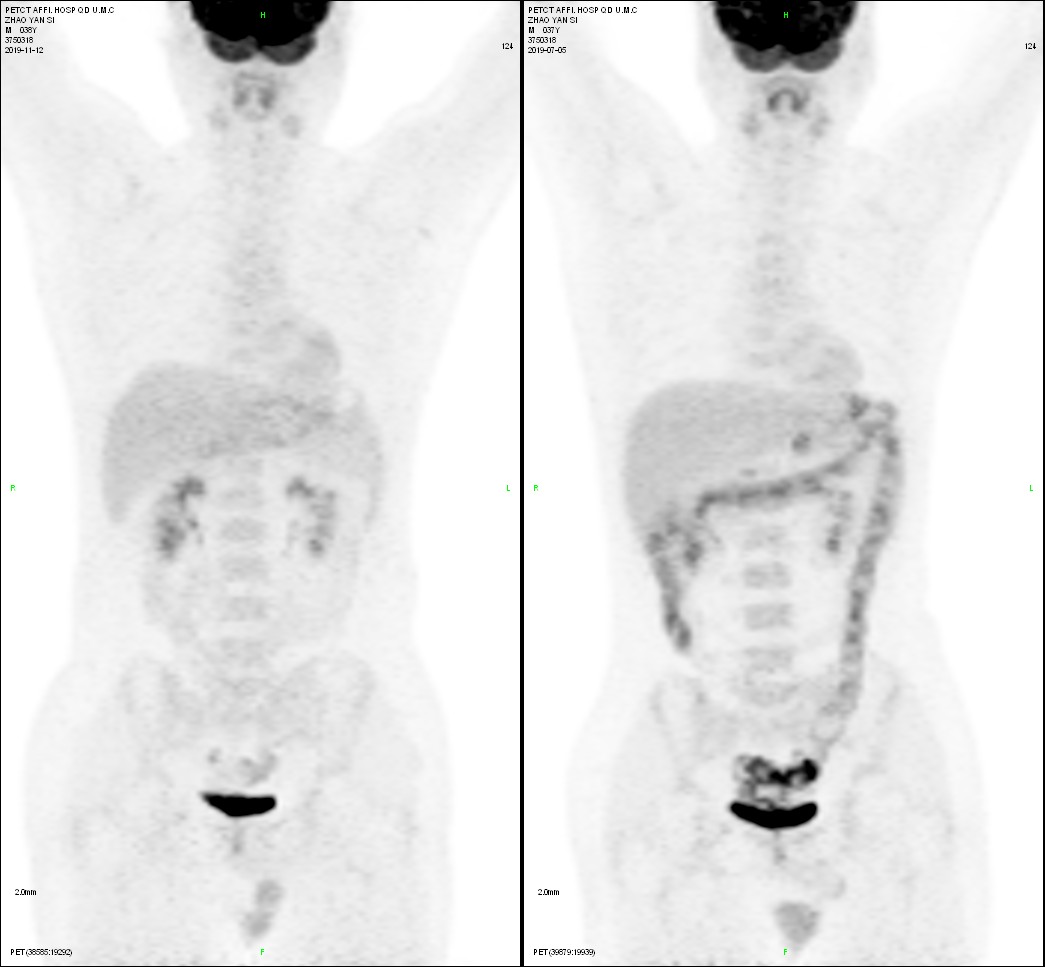


After the treatment Before the treatment

**(A)** PET-CT scanning panorama.


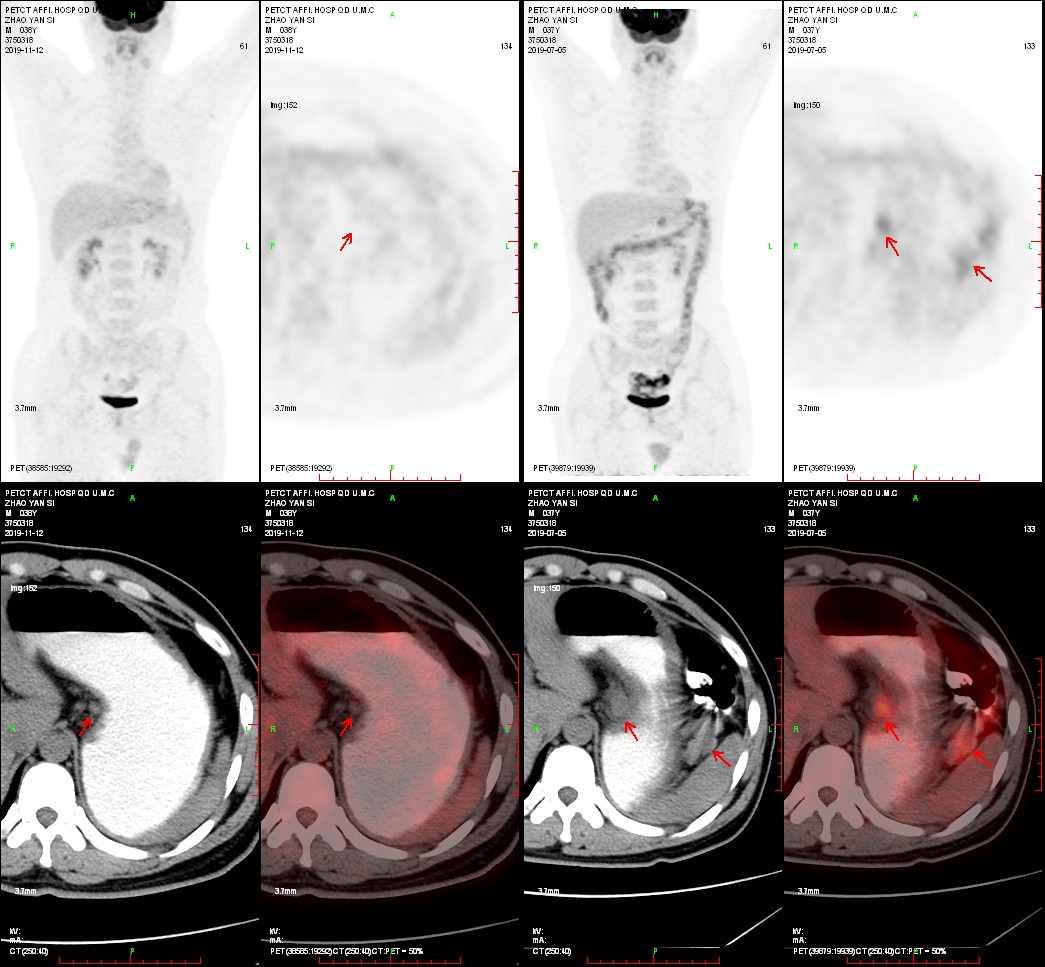

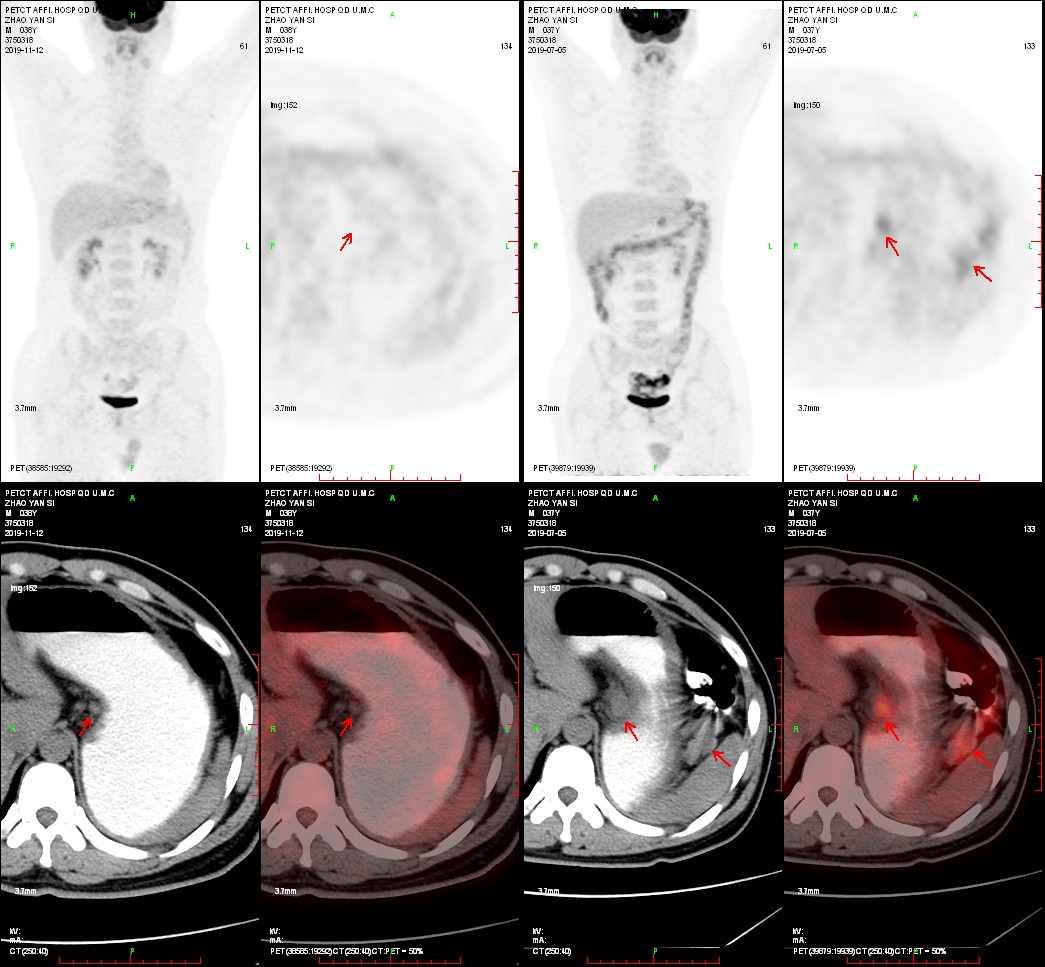


After the treatment Before the treatment

**(B)** The PET-CT showed the patient with an uneven thickening of gastric cardia and gastric fundus wall, the max SUV was evaluated as 25.0 before the therapy of toripalimab combined with anlotinib, and was significantly reduced with the metabolism reduced to SUV 4.8 after the treatment.


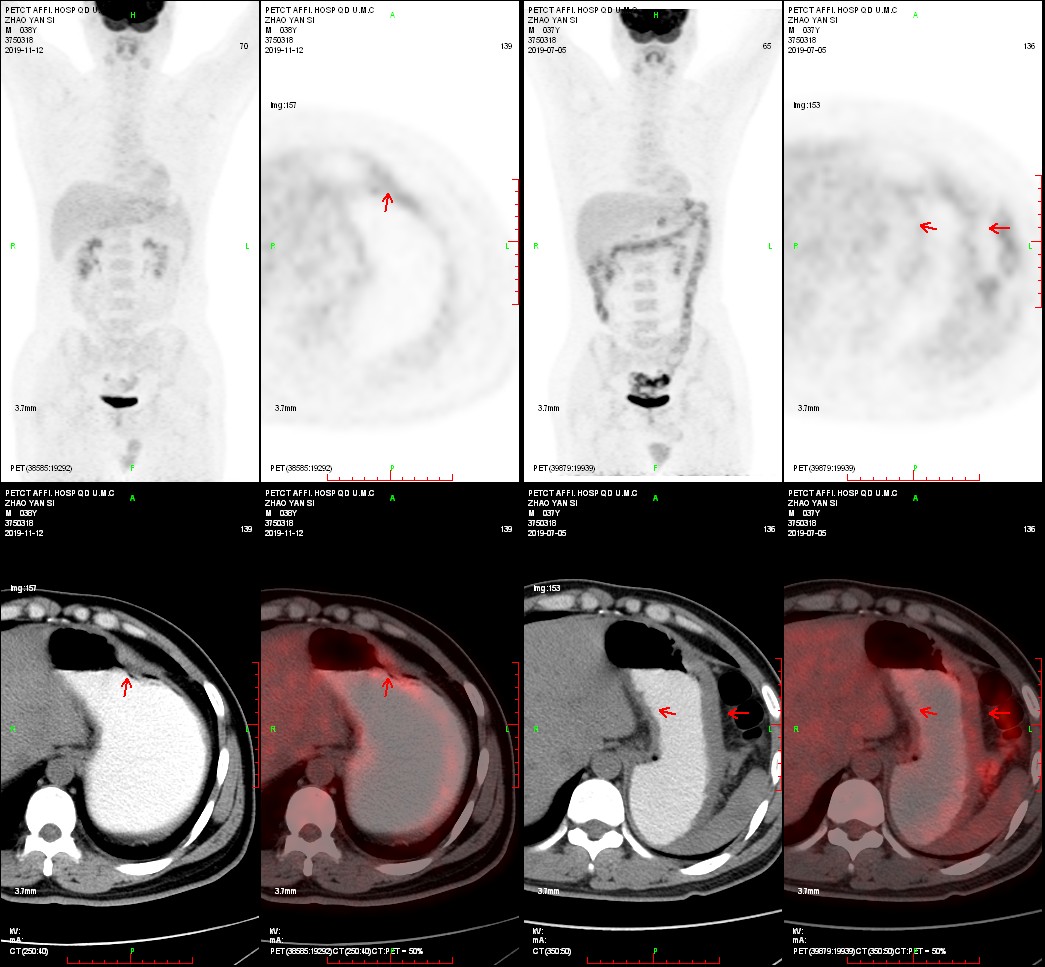

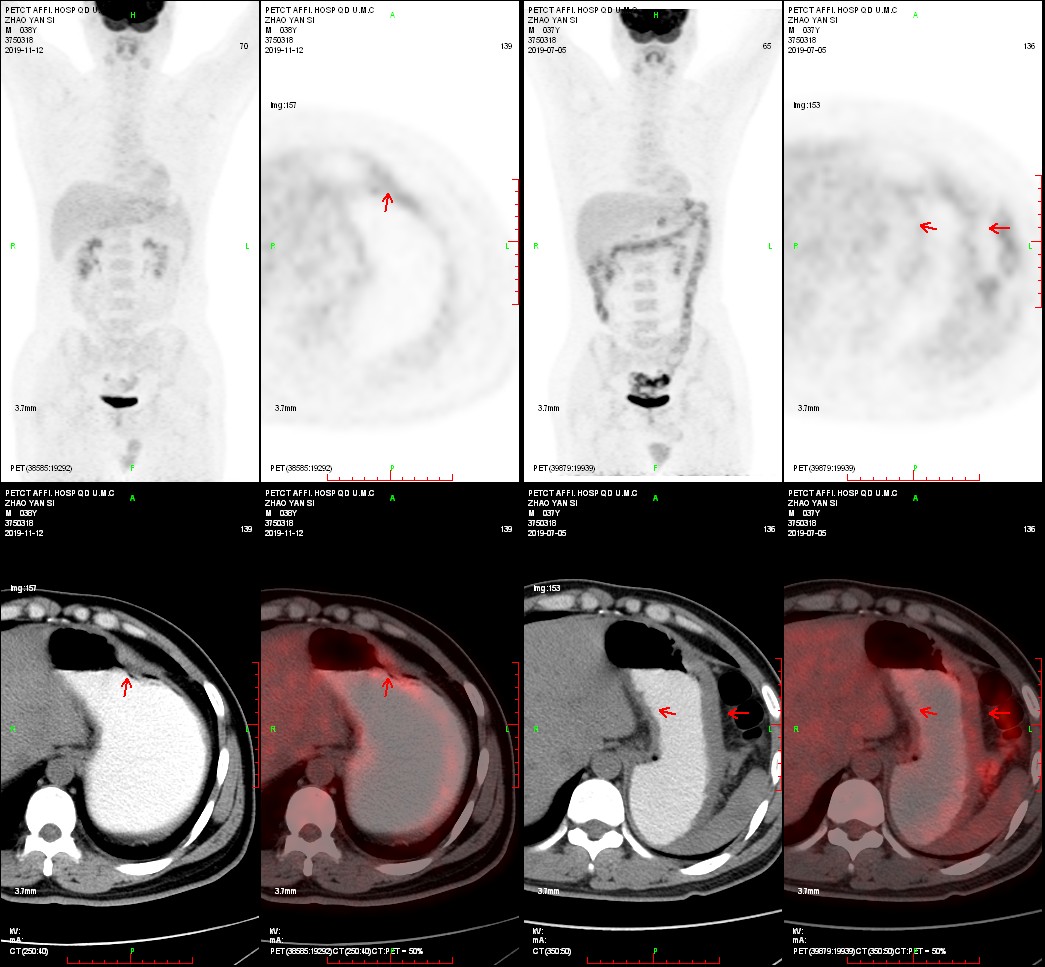


After the treatment Before the treatment

**(C)** The lymph nodes in hepatogastric space, posterior pancreas and abdominal aorta can be observed with the max SUV 3.2 before the therapy of toripalimab combined with anlotinib, and was significantly narrower without any abnormal metabolism after the treatment.


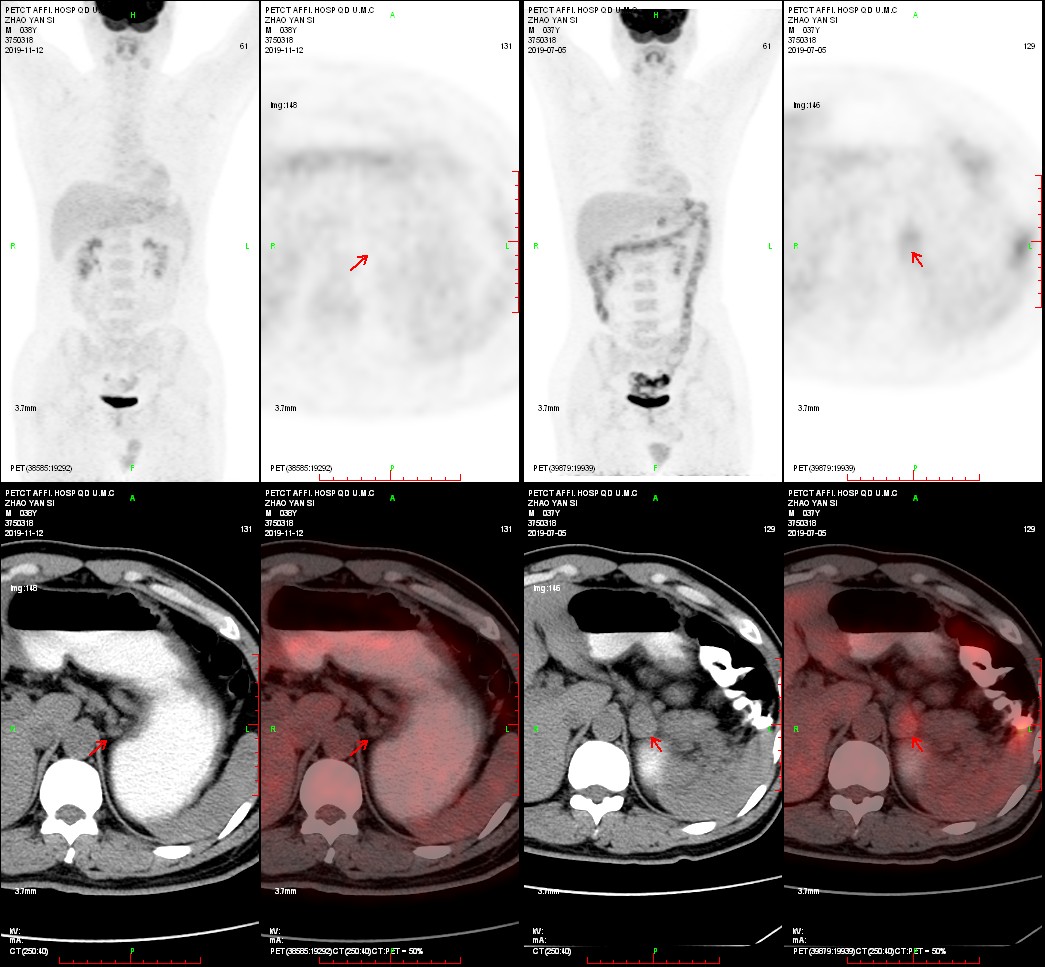


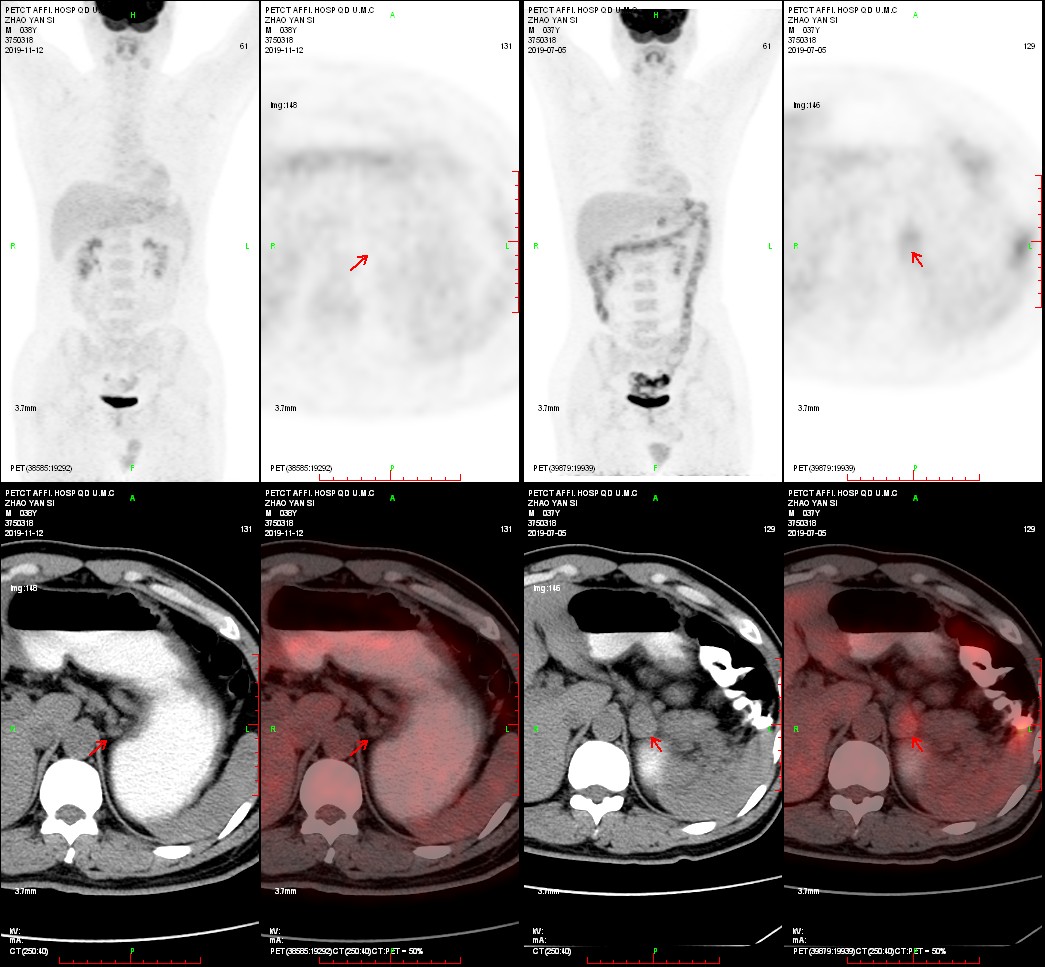


After the treatment Before the treatment

**(D)** Posterior pancreas and abdominal aorta can be observed with the max SUV 3.2 , accompanied by mediastinal lymph nodes enlargement before the therapy of toripalimab combined with anlotinib, and the metabolism of lymph nodes in mediastinum was reduced to SUV 1.9 without abnormality in the head of pancreas

**Supplementary Figure 2.** Ptient GC-18 who achieved PCR after 8 courses combination treatment of toripalimab and anlotinib, achieved the disease downstaging and under surgery after the treatment. **(1)** Gastroscopic pathology before the treatment; **(2)** Surgical pathology showed PCR; **(3)** PET-CT scan before and after 3 courses of the combination treatment: **(A)** Pet CT scanning panorama; **(B)** The PET-CT showed the patient with an uneven thickening of gastric cardia and gastric fundus wall, the max SUV was evaluated as 25.0 before the therapy of toripalimab combined with anlotinib, and was significantly reduced with the metabolism reduced to SUV 4.8 after the treatment; **(C)** The lymph nodes in hepatogastric space, posterior pancreas and abdominal aorta can be observed with the max SUV 3.2 before the therapy of toripalimab combined with anlotinib, and was significantly narrower without any abnormal metabolism after the treatment; **(D)** Posterior pancreas and abdominal aorta can be observed with the max SUV 3.2 , accompanied by mediastinal lymph nodes enlargement before the therapy of toripalimab combined with anlotinib, and the metabolism of lymph nodes in mediastinum was reduced to SUV 1.9 without abnormality in the head of pancreas.
