# Supplementary material for: Anlotinib Combined with Toripalimab as Second-Line Therapy for Advanced, Relapsed Gastric or Gastroesophageal Junction Carcinoma
Source: Oncologist. 2022 Jul 20;27(11):e856–69. doi: 10.1093/oncolo/oyac136 (PMC9632317; doi:10.1093/oncolo/oyac136)
Supplement: oyac136_suppl_Supplementary_Figure_S3 [file oyac136_suppl_supplementary_figure_s3.docx]

**(2)**

| Best Response | PD (n/5) | PR/CR(n/20) | Odds Ratio | P |
| --- | --- | --- | --- | --- |
|  |  |  | M-H, Fixed, 95% CI |  |
| Male | 3 | 13 | 0.81 [0.11, 6.04] | 0.84 |
| Surgery | 1 | 14 | 0.63[0.03, 5.78] | 0.92 |
| HP(+) | 2 | 11 | 0.55 [0.07, 4.01] | 0.55 |

**Supplementary Figure 3.** **(1)** The ORR of different characteristic subtype (Hp: Helicobacter pylori); **(2)** Characteristic analysis of enrolled patients; **(3)-(8)** Survival analysis of different characteristic subtype.
