# Supplementary material for: Anlotinib Combined with Toripalimab as Second-Line Therapy for Advanced, Relapsed Gastric or Gastroesophageal Junction Carcinoma
Source: Oncologist. 2022 Jul 20;27(11):e856–69. doi: 10.1093/oncolo/oyac136 (PMC9632317; doi:10.1093/oncolo/oyac136)
Supplement: oyac136_suppl_Supplementary_Tables [file oyac136_suppl_supplementary_tables.docx]

**Supplementary Table 1.** The trial information.

| Trial Information | |
| --- | --- |
| Disease | Gastric or esophagogastric junction cancer (GC/EGJC) |
| Stage of disease/ treatment | Metastatic/Advanced |
| Prior therapy | First-line chemotherapy (platinum and fluoropyrimidine doublet therapy) |
| Type of study | Open-label, Exploratory, Single-Arm |
| Primary endpoints | Objective response rate (ORR) and safety |
| Secondary endpoints | Median progression-free survival (mPFS) |
| Treatment | Anlotinib (12 mg/day, orally, 2 weeks of treatment followed by 1-week off-treatment) combined with toripalimab (240 mg, intravenous drip over 60 min, once every 3 weeks) |

**Supplementary Table 2.** Two levels of anlotinib dose adjustment.

| Dosage levels | Administration | Dosage |
| --- | --- | --- |
| 1-standard dose | 12mg po. qd | 12mg anlotinib capsule, one capsule |
| 2-reduced one dose | 10mg po. qd | 10mg anlotinib capsule, one capsule |
| 3-reduced one dose | 8mg po. qd | 8mg anlotinib capsule, one capsule |

po, oral; qd, one times per day

**Supplementary Table 3.** The recommended delay in medication administration and dosage changes when a therapy-related toxicity occurs.

| Adverse effect grade NCI CTCAE 5.0 | Drug administration | Dosage reduce |
| --- | --- | --- |
| Grade 0 to 2 | On time | No change |
| Grade 3 | Delay until adverse effect grade is lower than 2* | Reduce one dose level |
| Grade 4 | Delay until adverse effect grade is lower than 2* | Reduce one dose level. Terminate treatment if deemed necessary |

*If the delay dose not recover after 3 weeks, the treatment should be terminated permanently.

CTCAE, Common Terminology Criteria for Adverse Events; NCI, National Cancer Institute.

**Supplementary Table 4.** The recommended delay in medication administration and dosage changes when a decrease in platelet count occurs.

| Adverse effect grades | Dose adjusting protocol | Management |
| --- | --- | --- |
| Grade 1: PLT count 100 to 75 ×10^9^/L | Maintain the original dose | Follow-up regularly |
| Grade 2: PLT count 75 to 50 ×10^9^/L | Delayed until adverse effect grade lower to < Grade 2 in 2 weeks, resume original dose. | Repeat RBC in 2 to 3 days; recommend active treatment. RBC weekly during follow-ups. |
|  | Delayed until adverse effect grade lower to < Grade 2 in 3 weeks, lower one dose level. | Repeat RBC in 2 to 3 days; recommend active treatment. RBC weekly during follow-ups. |
| Grade 3: PLT count 50 to 25 ×10^9^/L | Delayed until adverse effect grade lower to < Grade 2 in 3 weeks, lower one dose level. | Repeat RBC in 2 to 3 days; recommend active treatment. RBC weekly during follow-ups. |
| Grade 4: PLT count < 25×10^9^/L | Terminate therapy | Daily RBC until adverse effect grade is ≤ Grade 2; actively give PLT transfusion |

PLT, platelet; RBC, red blood cell.

**Supplementary Table 5.** The recommended delay in medication administration and dosage changes after a bleeding event.

| Adverse effect grades | Dose adjusting protocol | Management |
| --- | --- | --- |
| Grade 1 | Maintain the original dose | Follow-up as planned |
| Grade 2 | Delayed until adverse effect grade lower to < Grade 2, lower one dose level | Active menagement |
| ≥Grade 3 | Terminate therapy | Emergent intervention |

**Supplementary Table 6.** The recommended delay in medication administration and dosage changes when liver function abnormalities (higher ALT, elevated AST or elevated total bilirubin) occur.

| Adverse effect grades | | Dose adjusting protocol | Management |
| --- | --- | --- | --- |
| Grade 1 | | Maintain the original dose | Follow-up planned |
| Grade 2 | Normal baseline | Delayed until adverse effect grade lower to < Grade 2 in 3 weeks, lower one dose level | Activity reserve and monitor hepatic function weekly |
|  | Abnormal baseline | Maintain the original dose | Activity reserve and monitor hepatic function weekly |
| Grade 3 | | Delayed until adverse effect grade lower to < Grade 2 in 3 weeks, lower one dose level | Actively reserve and monitor liver function twice weekly until adverse effect lower to < Grade 2 or can be explained |
| Grade 4 | | Terminate therapy | Actively reserve and monitor liver function twice weekly until adverse effect lower to < Grade 2 or can be explained |

ALT, ananine aminotrasferase; AST, aspartate aminotransferase.

**Supplementary Table 7.** The recommended delay in medication administration and dosage changes when proteinuria occurs.

| Adverse effect grade | Drug administration | Management |
| --- | --- | --- |
| Grade 1: UA shows protein + or 24 hours protein quantity < 1g | Maintain the original dose | Follow-up as planned |
| Grade 2: UA shows protein + or 24 hours protein quantity > 1g and <2g | Maintain the original dose | Active treatment with weekly UA; consult nephrologist when necessary |
| Grade 2: UA shows protein + or 24 hours protein quantity > 2g and <3.5g | Delayed until adverse effect grade lower to Grade 2 in 3 weeks ,lower one dose level | Active treatment; consult nephrologist when necessary, terminate treatment when experience adverse effect for the third time |
| Grade 3: UA shows protein + or 24 hours protein quantity ≥3.5g | Delayed until adverse effect grade lower to Grade 2 in 3 weeks ,lower one dose level | Active treatment; consult nephrologist when necessary, terminate treatment when experience adverse effect for the third time |

UA, urinalysis.

**Supplementary Table 8.** The possible evaluation of the causal relationship between adverse events and trial medications.

|  | **1** | **2** | **3** | **4** | **5** |
| --- | --- | --- | --- | --- | --- |
| Definitely relevant | **+** | **+** | **+** | **+** | **+** |
| Very likely | **+** | **+** | **+** | **+** | **?** |
| Possible related | **+** | **+** | **±** | **±** | **?** |
| May not be relevant | **+** | **--** | **±** | **±** | **?** |
| Definitely irrelevant | **--** | **--** | **--** | **--** | **--** |

?,possibly related; --, negative; +, positive; **±**, hard to be positive or negative.
